# Mutations in CIC and IDH1 cooperatively regulate 2-hydroxyglutarate levels and cell clonogenicity

**DOI:** 10.18632/oncotarget.2401

**Published:** 2014-08-27

**Authors:** Suganthi Chittaranjan, Susanna Chan, Cindy Yang, Kevin C. Yang, Vincent Chen, Annie Moradian, Marlo Firme, Jungeun Song, Nancy E. Go, Michael D. Blough, Jennifer A. Chan, J. Gregory Cairncross, Sharon M. Gorski, Gregg B. Morin, Stephen Yip, Marco A. Marra

**Affiliations:** ^1^ Canada's Michael Smith Genome Sciences Centre, BC Cancer Agency, Vancouver, BC, Canada; ^2^ California Institute of Technology, Beckman Institute, Pasadena, CA, USA; ^3^ Department of Molecular Biology and Biochemistry, Simon Fraser University, Burnaby, BC, Canada; ^4^ Department of Clinical Neurosciences, University of Calgary, Calgary, AB, Canada; ^5^ Southern Alberta Cancer Research Institute, University of Calgary, Calgary, AB, Canada; ^6^ Department of Pathology & Laboratory Medicine Vancouver General Hospital, Vancouver, BC, Canada; ^7^ Department of Pathology and Laboratory Medicine, University of Calgary, Calgary, AB, Canada; ^8^ Clark H. Smith Brain Tumour Centre, University of Calgary, Calgary, AB, Canada; ^9^ Department of Medical Genetics, University of British Columbia, Vancouver, BC, Canada

**Keywords:** CIC, IDH1, ACLY, citrate, 2HG, Oligodendroglioma

## Abstract

The majority of oligodendrogliomas (ODGs) exhibit combined losses of chromosomes 1p and 19q and mutations of isocitrate dehydrogenase (IDH1-R132H or IDH2-R172K). Approximately 70% of ODGs with 1p19q co-deletions harbor somatic mutations in the *Capicua Transcriptional Repressor* (*CIC*) gene on chromosome 19q13.2. Here we show that endogenous long (CIC-L) and short (CIC-S) CIC proteins are predominantly localized to the nucleus or cytoplasm, respectively. Cytoplasmic CIC-S is found in close proximity to the mitochondria. To study wild type and mutant CIC function and motivated by the paucity of 1p19q co-deleted ODG lines, we created HEK293 and HOG stable cell lines ectopically co-expressing CIC and IDH1. Non-mutant lines displayed increased clonogenicity, but cells co-expressing the mutant IDH1-R132H with either CIC-S-R201W or -R1515H showed reduced clonogenicity in an additive manner, demonstrating cooperative effects in our assays. Expression of mutant CIC-R1515H increased cellular 2-Hydroxyglutarate (2HG) levels compared to wild type CIC in IDH1-R132H background. Levels of phosphorylated ATP-citrate Lyase (ACLY) were lower in cell lines expressing mutant CIC-S proteins compared to cells expressing wild type CIC-S, supporting a cytosolic citrate metabolism-related mechanism of reduced clonogenicity in our *in vitro* model systems. ACLY or phospho-ACLY were similarly reduced in CIC-mutant 1p19q co-deleted oligodendroglioma patient samples.

## INTRODUCTION

Oligodendrogliomas (ODG)s are a histologically, therapeutically and cytogenetically distinct subgroup of gliomas [1]. The majority of ODGs exhibit combined loss of chromosomes 1p and 19q and recurrent mutations of IDH1 or IDH2 (R132H or R172K respectively) [2]. Such ODGs are slow growing, respond well to chemotherapy and patients exhibit improved survival outcome compared to other gliomas that retain 1p [3, 4]. DNA sequencing of ODG tumour samples with 1p and 19q deletions identified somatic mutations in the gene *CIC* [2, 5] located on chromosome 19q13.2. *CIC* mutations affected 53-69% of ODG cases, and included missense, nonsense, and out-of-frame insertions and deletions [2, 5-7]. More than 25% of the *CIC* mutations were predicted to inactivate their encoded proteins [5]. Such inactivating mutation patterns are generally observed in tumour suppressor genes such as *TP53* and *FBXW7,* but not in oncogenes [5]. Among the somatic mutations observed in CIC, 41% and 7% of the missense mutations were found in the *CIC* DNA-binding HMG domain and the Groucho protein-protein interaction domain, respectively [2, 5, 7, 8].

Somatic mutations were also observed in the gene encoding far upstream element (FUSE) binding protein1 (*FUBP1*) on chromosome 1p31.1 [5] at a frequency (16%) lower than CIC mutations. A recent study of 45 ODGs with 1p19q co-deletions identified two hotspot point mutations (-124 and -146 bp upstream from the ATG start site) in the promoter region of telomerase reverse transcriptase (TERT) [9] on chromosome 5p15.33, the catalytic subunit of the enzyme telomerase. These point mutations create a binding sequence for the ETS transcription factor, which can subsequently activate *TERT* transcription. Exactly how these mutations contribute to oligodendrogliomagenesis has yet to be determined.

*CIC* is well conserved across evolutionary boundaries [10, 11], and was first identified as a HMG-box repressor downstream of the RTK-Ras–Raf–MAPK cascade [12] in *Drosophila*. In addition to terminal and dorsoventral patterning [12, 13] and wing vein specification [14], *CIC* is involved in ommatidial cell proliferation [15]. During ommatidial development, activation of EGFR signaling and down regulation of CIC levels was required for promoting cell growth and cell proliferation. At least two main CIC protein isoforms, which differ in both size (short form, CIC-S; long form, CIC-L) and in their N-terminal regions, have been identified in *Drosophila* and mammals. Both CIC-S and CIC-L are highly conserved between mouse and human (>99% amino acid identity) [10, 11], with predicted lengths of 1,608 AA and 2,517 AA respectively.

Relatively few studies have addressed the role of CIC in human biology and disease. For example, CIC appears to repress the PEA3 family of ETS transcription factors in cancers. Rare cases of Ewing's sarcoma express a novel CIC–DUX4 fusion protein encoded by a recurrent chromosomal translocation t(4;19)(q35;q13) [16]. This fusion protein activated transcription of the PEA3 family genes *ERM/ETV5* and *ETV1* and overexpression of PEA3 family proteins was associated with invasive and metastatic phenotypes in breast and gastric cancers and in rhabdomyosarcoma [17]. In HEK293 cells, ribosomal protein S6 kinase II (p90RSK) phosphorylated CIC and promoted the binding of phosphorylated CIC to 14-3-3 regulatory proteins [18]. This interaction reduced the binding of CIC to CIC binding “TGAATGAA” promoter sequences and reduced CIC repressor activity. The reduced binding of CIC correlated with increased expression of CIC targets *in vitro*, such as ETV5 [18]. Finally, association of wild type CIC with polyglutamine-expanded ATAXIN-1 results in neurotoxicity and contributes to spinocerebellar ataxia (SCA1) [10, 19]. An ATXN1-CIC protein complex showed enhanced transcriptional repressor activity compared to CIC alone [10] and reducing CIC levels rescued the Ataxia phenotype in mouse models period. Additional *in vitro* and *in vivo* studies in mammalian systems are now required to elucidate the cellular functions of both CIC mutant and wild type proteins.

Alterations in glycolysis and citrate metabolism contribute to the survival of cancer cells including gliomas [20-22]. In cancers cells, the citrate transporter SLC25A1 preferentially transports mitochondrial citrate produced by the TCA cycle to the cytosol, where citrate plays a central role in metabolism [23, 24]. Cytosolic citrate can be converted to oxaloacetate (OAA) and acetyl-CoA by the enzyme ATP-citrate lyase (ACLY) in an ATP dependent manner. Acetyl-CoA is required for *de novo* lipid synthesis and acetylation of histones in proliferating cancer cells [24, 25]. Cytosolic citrate is also converted into isocitrate by acotinase and then into α-2-ketoglutarate (2KG) by IDH1 [24]. However, mutant variants of IDH1 (eg.R132H/C/S/L/G/V) exhibit a neomorphic function that converts 2KG to the oncometabolite (R)-2 hydroxyglutarate (2HG) [26, 27]. Intracellular levels of 2HG are high in cancer cells containing IDH mutations and are sufficient to promote cell transformation [28]. In gliomas, 2HG significantly decreased 5-hydroxymethylcytosine (5hmC), increased DNA methylation and reduced DNA de-methylation, ultimately leading to a CpG island methylator phenotype (CIMP) [29].

In 1p19q co-deleted ODG, *CIC* mutations co-occur with mutations in either IDH1 or IDH2 in approximately 53-69% of cases, but the functional consequences of this co-occurrence are unknown [2, 5-7]. Here we describe, for the first time, the sub-cellular localization of endogenous CIC isoforms in human cells, including ODG cells with 1p19q co-deletions. Endogenous CIC-L was predominantly localized to the nucleus. Endogenous CIC-S was predominantly cytoplasmic, in close proximity to mitochondria, and formed complexes with ACLY which synthesizes acetyl-CoA in the cytosol. We show that cells expressing mutant CIC proteins had lower levels of active phosphorylated ACLY (pACLY) compared to cells expressing wild type CIC. Cells co-expressing mutant IDH1-R132H and mutant CIC-R1515H displayed increased 2HG levels compared to cells co-expressing mutant IDH1-R132H and wild type CIC. Cells expressing IDH1-R132H mutations exhibited reduced cell proliferation compared to cells expressing wild type IDH1 or cells co-expressing both wild type CIC and IDH1. Co-expression of mutant IDH1-R132H and wild type CIC partially rescued the reduction in cell proliferation. Co-expression of mutant CIC (-R1515H and -R201W) and mutant IDH1-R132H further reduced clonogenicity compared to cells expressing mutant IDH1-R132H. Our data provide the first insights into the localization and function of mammalian wild type CIC and the mutant CIC proteins found in ODGs (eg. CIC-R1515H and CIC-R201W) in wild type IDH1 and mutant IDH1-R132H backgrounds and allude to novel non-nuclear functions of CIC.

## RESULTS

### Capicua isoforms localize to different cellular compartments in mammalian cells

**Figure 1:**
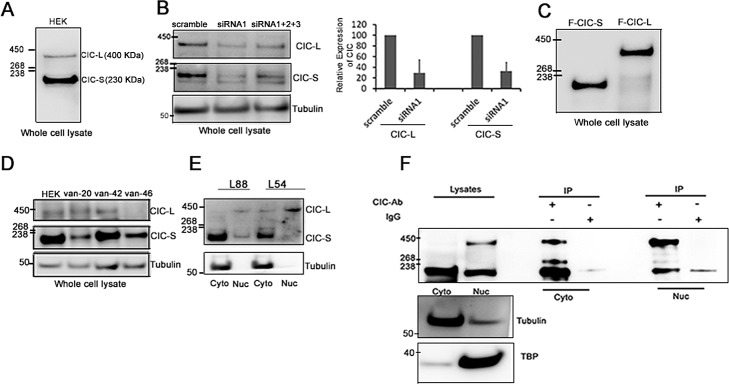
Subcellular localization of endogenous CIC isoforms in mammalian cells A. Western blots using an anti-CIC antibody detected CIC isoforms CIC-L (400 KDa) and CIC-S (250 KDa) in HEK cells. B. HEK cells were transfected with a single siRNA (siRNA1) or a three siRNAs in combination (siRNA1+2+3) against CIC. Reductions in CIC-S and CIC-L were detected after 72hrs using western blots. Data are representative of three independent experiments. Changes in band intensities for CIC-L and CIC-S after siRNA1 treatment relative to scramble siRNA were quantified using densitometry and averaged over three independent experiments to generate the bar graph. C. N-terminal FLAG fusions with CIC-S (F-CIC-S) and CIC-L (F-CIC-L) were ectopically expressed in HEK cells and detected using a FLAG antibody. D. Whole lysate preparations of gliomas (van-20: Glioblastoma, no IDH1 mutation, no 1p19q co-deletion; van-42 and van 46: oligodendroglioma, IDH1_R132H mutation, 1p19q loss) were probed for endogenous CIC expression using an anti-CIC antibody. E. Subcellular fractionations of ODG cell lines BT054 and BT088 were performed and cytoplasmic (Cyto) and nuclear (Nuc) fractions were isolated. Endogenous CIC isoforms were detected using western blots. Data shown are representative of three independent experiments. CIC-S is enriched in cytoplasmic fractions and CIC-L is enriched in nuclear fractions. F. Cytoplasmic (Cyto) and nuclear (Nuc) fractions were isolated from HEK cells and endogenous CIC isoforms were isolated using immunoprecipitations (IPs) with an anti-CIC antibody. Subcellular localization of the isoforms was detected using a different anti-CIC antibody (by western blot analysis). Tubulin (Cyto) and TATA binding protein (TBP; Nuc) were detected as control proteins. Data shown are representative of at least three independent experiments.

To further characterize the predominant endogenous CIC protein isoforms (CIC-L and CIC-S) in human cells [2], we prepared whole cell lysates of human embryonic kidney cell line HEK-293A: (hereafter referred to as HEK) and detected CIC proteins using western blotting. Our analysis of primary amino acid sequences predicted that CIC-S and CIC-L were 164 KDa and 259 KDa in size. However, an anti-CIC antibody (Methods) consistently detected two major protein bands that migrated at approximately 230 KDa and 400 KDa (Figure 1A; Supplementary Figure S1). We occasionally detected a smaller isoform at 170 KDa (Supplementary Figure S1) but did not study this minor isoform further due to its variable presence. To validate the CIC antibody, we knocked down *CIC* using three different siRNAs (Methods) and quantitated CIC proteins using western blots and densitometry. Compared to scramble siRNA controls, *CIC*-siRNA1 or *CIC*-siRNAs1+2+3 significantly reduced both 230 KDa CIC-S and 400 KDa CIC-L protein bands to approximately 15% and 45% of control levels respectively (Figure 1B). To confirm the migration of CIC-S and CIC-L at 230 KDa and 400 KDa on protein gels, we ectopically expressed N-terminally FLAG fused CIC-S (F-CIC-S) and CIC-L (F-CIC-L) proteins in HEK cells and detected CIC using a FLAG antibody and western blotting. F-CIC-S and F-CIC-L were detected at approximately 230 KDa and 400 KDa respectively (Figure 1C), confirming that CIC-S and CIC-L migrate at these molecular weights. We next explored whether these isoforms were present in gliomas. We isolated proteins from one non-ODG tumour with a 1p deletion, two ODG tumors with 1p19q co-deletions, and cultured ODG cells BT054 (IDH1^+/R132H^/CIC^+/−)^ and BT088 (IDH1^+/+^/CIC^R1515H/−)^ [30], both containing 1p19q co-deletions. We detected the CIC-S isoform in all samples and the CIC-L isoform in two of three glioma tumor samples and in both ODG cell lines, indicating CIC isoforms of the expected size are present in glioma cells (Figure 1D-E, Supplementary Figure S1).

To determine the subcellular localization of endogenous CIC-S and CIC-L isoforms in HEK cells, we fractionated cells, purified cytoplasmic and nuclear fractions, and detected proteins that were enriched in these fractions (Methods). To assess the distribution of CIC-S and CIC-L, we immunoprecipitated (IP) CIC proteins from cytoplasmic and nuclear fractions and analyzed the IP samples using western blots. In fractionated samples, CIC-S at 230 KDa and CIC-L at 400 KDa were enriched in the cytoplasmic (59+/5%) and nuclear fractions (89+/−5%), respectively (Figure 1F). In ODG lines BT054 and BT088, CIC-S and CIC-L were also predominantly observed in the cytoplasmic and nuclear fractions, respectively (Figure 1E).

**Figure 2:**
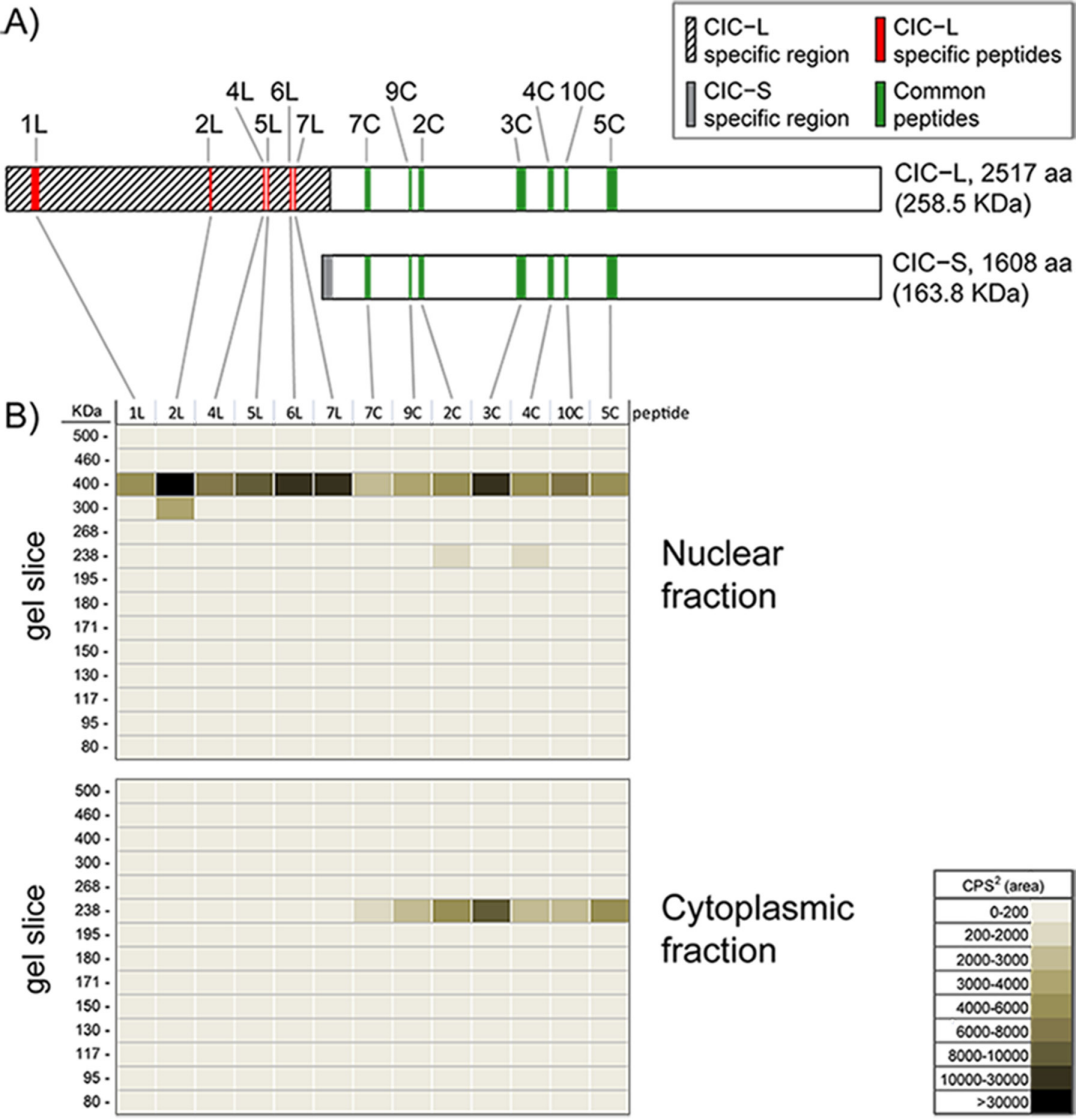
Mass spectrometry analysis confirms the localization of CIC-L to nuclei and CIC-S to the cytoplasm A. Shown are the locations of the CIC peptides used to monitor the subcellular location of CIC by Multiple Reaction Monitoring (MRM) MS assays. A protein alignment cartoon shows the positions of the measured peptides specific for CIC-L (1L-7L; red) and the measured peptides common for both isoforms (2C-10C; green), see Supplementary Figure 2 for the sequence of the peptides. CIC-L and CIC-S specific regions are indicated in hatched and grey, respectively. Unfilled regions are common to both isoforms. B. Nuclear and cytoplasmic fractions were isolated from HEK cells and CIC isoforms were immunoprecipitated using an anti-CIC antibody. Immunoprecipitates were run on a protein gel and proteins were isolated from 14 different slices of the gel in the 80-500 KDa region. Isolated proteins were used to detect peptides specific to CIC isoforms by MRM-MS. Peptides from each slice were measured. The peptide ion densities for the peptides are shown as a heat map for nuclear and cytoplasmic fractions (summed areas of each peptide transition peak, as counts per second^2^ (cps^2^)). The data are representative of two independent trials.

To confirm the subcellular localization of CIC isoforms, we designed Multiple Reaction Monitoring (MRM) mass spectrometry (MS) assays for 10 peptides common to both isoforms and 9 peptides unique to the CIC-L isoform only (Figure 2A, Supplementary Figure S2). CIC protein IPs from cytoplasmic and nuclear fractions were separated on Tris-Acetate gels, and CIC peptide levels were measured in 14 gel slices spanning the 80-500 KDa range (Methods). In the nuclear fraction, 6/9 peptides unique to CIC-L and 7/10 peptides common to both isoforms were detected only at 400 KDa (Figure 2B), suggesting these peptides originated from the CIC-L isoform. In the cytoplasmic fraction at 230 KDa, we detected 7/10 peptides common to both isoforms but no peptides unique to CIC-L were observed. These data are consistent with the notion that the peptides detected at 230 KDa in the cytoplasmic fraction originated from the CIC-S isoform (Figure 2C). Taken together, these results confirmed that the proteins we detected at 230 and 400 KDa were indeed CIC proteins, and that CIC-S is predominantly localized to the cytoplasm, while CIC-L is predominantly localized to the nucleus.

### Cytoplasmic CIC-S is localized to mitochondria

**Figure 3:**
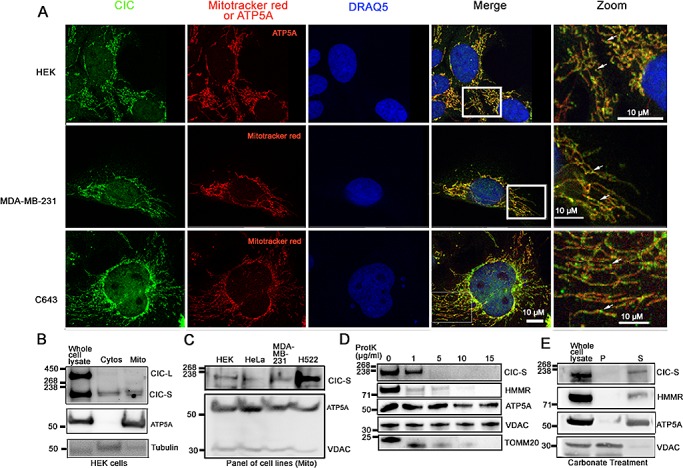
CIC-S is in close proximity to mitochondria and present in both cytosolic and mitochondrial fractions A. To further study the subcellular localization of CIC, an immunofluorescence assay was performed using an anti-CIC antibody (green). Mitochondria were visualized using either Mitotracker red or an anti-ATP5A antibody (red). The nucleus was visualized using DRAQ5 stain (blue). HEK, MDA-MB-231 (breast carcinoma) and C643 (thyroid carcinoma) cells were observed using a confocal microscope. Boxes in the merge column indicate the zoomed area. Arrows in magnified images (labeled Zoom) show CIC-positive-puncta localizing along mitochondria. B. HEK cytosolic (Cytos) and mitochondrial (Mito) fractions were further isolated from cytoplasmic fractions and CIC-S was detected using western blots. To validate the fractionation method, control proteins tubulin (cytos) and ATP5A (mito) were also detected. CIC-S is enriched in the cytosolic and mitochondrial fractions. Mitochondrial fractions were isolated from human cancer cell lines HeLa (ovarian), MDA-MB-231 (breast) and H522 (lung) and CIC-S was detected using an anti-CIC antibody and western blots. Control proteins ATP5A and VDAC (mitochondrial) were detected. CIC-S was found in mitochondrial fractions in all cells. C. The mitochondrial fraction was isolated from HEK cells and a Proteinase K protection assay was performed using 0-15μg/ml Proteinase K (ProtK). CIC-S protein was detected following treatment with Proteinase K. Control proteins VDAC (embedded in the mitochondrial outer membrane), ATP5A (mitochondrial matrix), TOMM20 (translocase of outer mitochondrial membrane), and HMMR (loosely associated with the outer mitochondria membrane) are also shown. ATP5A and VDAC but not CIC-S, TOMM20 and HMMR were protected at 15μg/ml ProtK indicating that CIC-S is localized outside mitochondria. Results shown are representative of three independent experiments. D. Mitochondrial fractions from HEK cells were treated with carbonate buffer and centrifuged to pellet (P) proteins embedded in the mitochondrial membrane fraction. The supernatant (S) contained proteins located in the inner membrane space, matrix and the proteins loosely bound to mitochondrial outer membrane. CIC-S was detected in the supernatant suggesting it is not embedded in the mitochondrial membrane. The result shown is representative of three experimental replicates.

During terminal and dorsoventral patterning of *Drosophila* embryos, CIC acts as a transcriptional repressor [12], yet in our standard culture conditions (Methods), ~59% of CIC-S was found in the cytoplasm. This indicated to us the potential for CIC functions beyond transcriptional regulation. To further explore the localization of CIC-S, we detected cytoplasmic CIC using immunofluorescence (IF). We visualized CIC in HEK, MDA-MB-231 (breast carcinoma), C643 (thyroid carcinoma), H522 (lung carcinoma), and HeLa (ovarian carcinoma) cells using an anti-CIC antibody that can detect both CIC-S and CIC-L. Consistent with our nuclear and cytoplasmic fractionation studies, we observed CIC in both the nucleus and cytoplasm (Figure 3A). An unexpected observation was that cytoplasmic CIC signal was concentrated in puncta apparently close to mitochondria (representative images in Figure 3A).

To determine whether CIC was localized to mitochondria, we examined subcellular fractions of HEK cells enriched for cytoplasmic organelles (includes mitochondria; hereafter referred to as the mitochondrial fraction) or non-organelle cytosolic material. Control proteins ATP5A (mitochondrial) and tubulin (cytosolic) were predominantly in the mitochondrial fraction and cytosolic fractions, respectively, as expected (Figure 3B). The CIC-S form was consistently observed in both cytosolic and mitochondrial fractions (Figure 3B) and CIC-S was also detected in the mitochondrial (Figure 3C) fractions in other carcinoma cell lines.

To explore the nature of the CIC-S association with mitochondria, we isolated the mitochondrial fraction from HEK cells and subjected it to increasing concentrations of Proteinase K (0-15 μg/ml) under isotonic conditions. Under these conditions, Proteinase K digests proteins exterior to the mitochondrial outer membrane. However, proteins embedded within the outer membrane and proteins located inside the membrane are protected from digestion. Mitochondrial proteins ATP5A (matrix), VDAC (embedded in the outer membrane), TOMM20 (translocase located outside of outer mitochondrial membrane) and RHAMM/HMMR, a protein loosely bound to mitochondria [31], served as controls. While mitochondrial proteins ATP5A and VDAC were protected from Proteinase K treatment at 15 μg/ml, CIC-S, TOMM20 and RHAMM proteins were not protected (Figure 3D), indicating that CIC-S was most likely localized outside the mitochondrial outer membrane. To confirm that CIC-S was located exterior to the outer membrane of mitochondria and not embedded in the outer membrane, we used carbonate to solubilize proteins that are not embedded in the outer or inner membrane of mitochondria [31]. We treated the mitochondrial fraction from HEK cells with carbonate buffer (Methods) and detected CIC-S using western blots. As shown in Figure 3E, CIC-S and matrix protein ATP5A solubilized in the carbonate buffer and VDAC remained in the pellet containing mitochondrial membrane. Our result indicates that CIC is not embedded in the mitochondrial membrane but may instead be loosely associated with the mitochondrial membrane (Figure 3E). These findings are consistent with the notion that in mammalian cells, endogenous CIC-S is located very near or perhaps on the surface of mitochondria.

**Figure 4:**
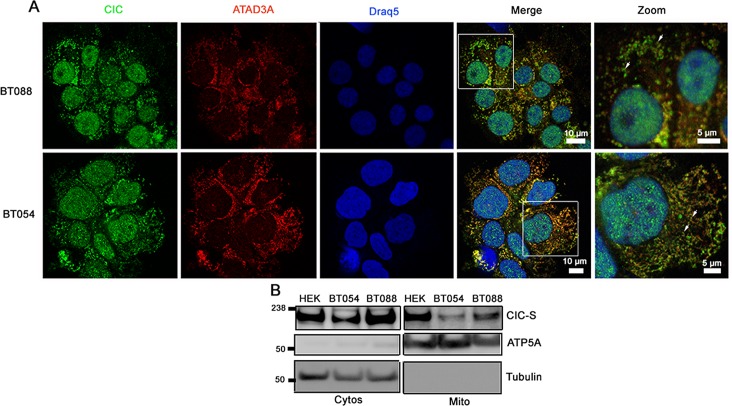
CIC-S localizes to mitochondria in 1p19q co-deleted oligodendroglioma cell lines A. Subcellular localization of CIC in ODG cell lines BT088 and BT054 was detected using immunofluorescence. CIC was detected using an anti-CIC antibody and mitochondria were stained using an anti-ATAD3A antibody. Cells were visualized using confocal fluorescence microscopy. B. Cytosolic (Cytos) and mitochondrial (Mito) fractions of BT054 and BT088 were isolated and CIC-S was detected using western blots. Control proteins ATP5A (Mito) and Tubulin (Cytos) were also detected.

We next sought to determine the subcellular localization of both wild type and mutant endogenous CIC in ODG cell lines (BT054 and BT088) that carried 1p19q co-deletions in different IDH1 genetic backgrounds. While BT054 carries mutant *IDH1* (R132H) and wild type *CIC* alleles, BT088 lacks mutant *IDH1* but carries a mutant *CIC* allele, encoding R1515H in the protein–protein interaction domain, and no wild type *CIC* allele [2, 30]. We performed immunofluorescence (IF) experiments using an anti-CIC antibody that recognized both wild type and mutant CIC and an antibody that recognizes the mitochondrial protein ATAD3A [32]. Cytoplasmic CIC appeared to be adjacent to mitochondria in both BT054 and BT088 ODG cell lines (Figure 4A).

To determine whether wild type and mutant CIC-S were detected in the mitochondrial fraction in ODG cells, we isolated cytosolic and mitochondrial fractions of both BT054 and BT088 and detected CIC using western blots. In both ODG cell lines, CIC-S was detected in both cytosolic and mitochondrial fractions (Figure 4B). Compared to wild type CIC in BT054, levels of CIC-R1515H in BT088 were observed at higher levels in both fractions, indicating CIC-R1515H is stable and that the mutation did not affect subcellular distribution between cytosolic and mitochondrial fractions.

### Expressions of both CIC and IDH mutant cooperatively increased 2HG production in HEK293 cells

**Figure 5:**
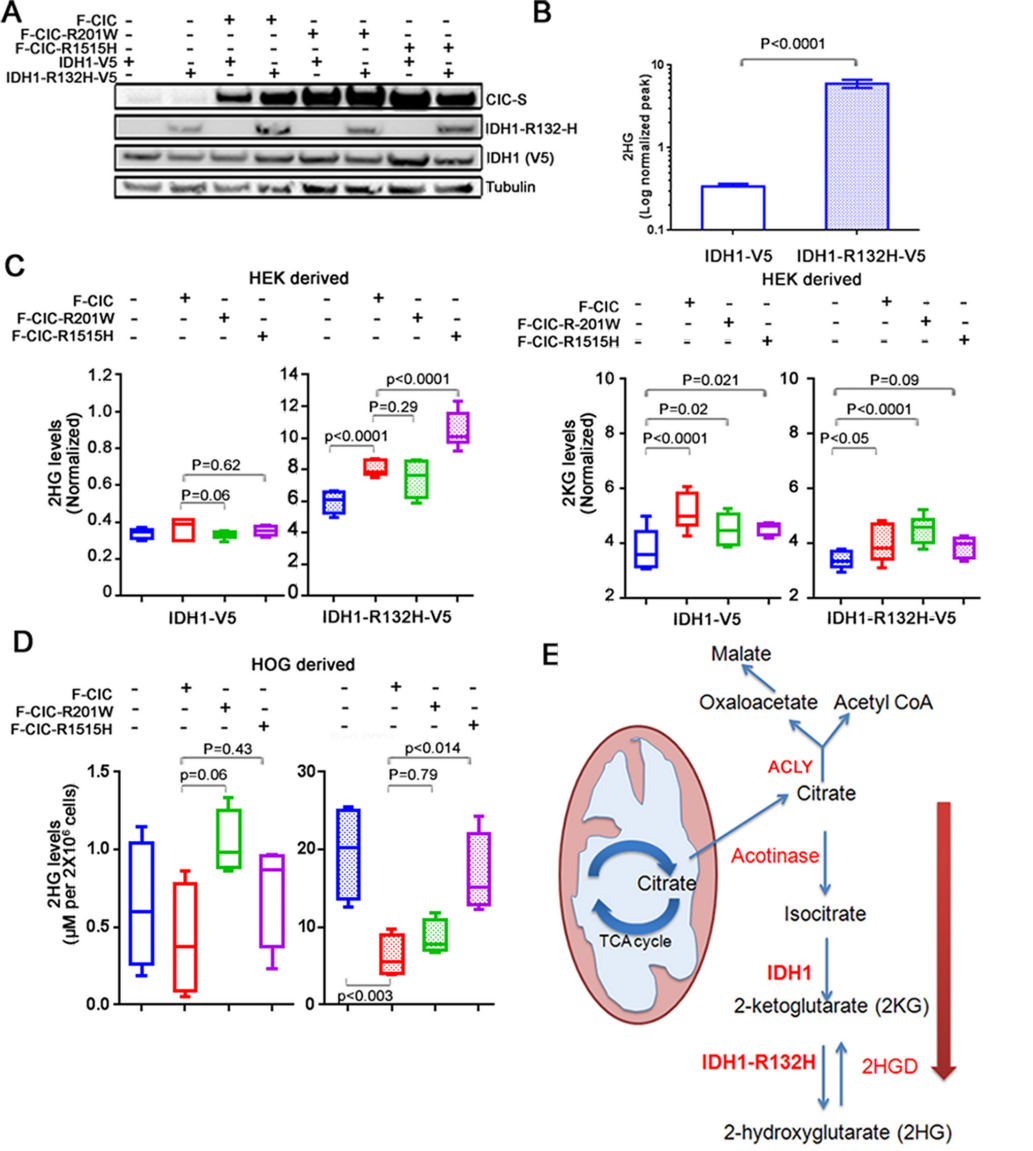
IDH1-R132H and mutant CIC coordinately regulate 2HG production *in vitro* A. Stable cell lines expressing either wild type IDH1-V5 or mutant IDH1-R132H-V5 in combination with wild type F-CIC or mutant F-CIC-R1515H or F-CIC-R201W were established and proteins detected using western blots. Expression of CIC and mutant IDH1-R132H were detected using antibodies specific to CIC or IDH1-R132H proteins. Mutant and wild type IDH1 proteins were fused to a V5 epitope and detected using an anti-V5 antibody. B. Levels of total cellular 2HG from HEK-derived stable cells (passage #11-14) expressing wild type IDH1-V5 or IDH1-R132H-V5 were detected using targeted MRM-mass spectrometry. The values reported are averaged peak areas for 2HG that were normalized to the total area within each biological replicate. Error bars represent the SD from 3 independent biological replicates and 3 technical replicates of each (3 MS runs of the same sample), for a total of 9 data points for each experiment. C. Levels of 2HG or 2KG were detected using targeted MRM-mass spectrometry from HEK-derived cells co-expressing wild type or mutant CIC with wild type or mutant IDH1 (passage #11-14). The values reported are averaged peak areas for 2HG or 2KG that were normalized to the total area within each biological replicate. Error bars represent the SD from 3 independent biological replicates and 3 technical replicates, for a total of 9 data points per experiment. Note the different scales of the Y-axes in the 2HG graphs. D. Levels of 2HG were detected using an enzymatic assay [37] from HOG-derived cells co-expressing wild type or mutant CIC with wild type or mutant IDH1 (passage #10-13). 2HG levels (μM) were calculated from standard curves established using D-2-Hydroxyglutaric acid. The averaged values reported were derived from four biological replicates. Note the different scales of the Y axes in the 2HG graphs. E. A model depicting the cooperative regulation of 2HG in cells co-expressing mutant IDH1-R132H and mutant CIC-R1515H/-201W. When high levels of citrate are produced in the mitochondria, citrate is shuttled to the cytosol. Cytosolic citrate may have two fates in cancer cells. (1) In actively proliferating cells, citrate is used by ACLY to produce Acetyl-CoA in an ATP dependent manner. Acetyl-CoA is used for *de novo* lipid synthesis. (2) Citrate is used to produce 2-alpha ketoglutarate (2KG) by IDH1. Mutant IDH1_R132H converts 2KG into 2-alpha-hydroxyglutarate (2HG). CIC is in close proximity to mitochondria and appears to play a role in diverting citrate towards 2HG production.

In our previous study [2], 69% of ODGs with 1p19q co-deletions carried mutations in both the *IDH* and *CIC* genes. Since mutations in IDH1 and IDH2 increase the production of 2HG [26] and endogenous CIC-S was observed close to mitochondria, we hypothesized that mutations in IDH and CIC-S might coordinately promote the production of 2HG. To test this hypothesis, we derived stable HEK cell lines that ectopically expressed wild type CIC-S or mutant CIC-S (both R1515H and R201W) fused to N-terminal FLAG (hereafter referred to as F-CIC or F-CIC-R1515H or F-CIC-R201W), in combination with wild type IDH1 (hereafter referred to as IDH1-V5) or mutant IDH1-R132H (hereafter referred to as IDH1-R132H-V5) fused to C-terminal V5 proteins (Figure 5A). We are aware of only two available 1p19q co-deleted ODG cell lines, described above. Both lines grow very slowly and are resistant to transfection. Hence, we developed and used stably transformed HEK cells, as such cells were reported to exhibit properties similar to those of immature neurons [33]. To confirm previous reports [26, 29] in which cells expressing IDH1-R132H-V5 exhibited higher levels of 2HG compared to cell lines expressing wild type IDH1, we first quantified the levels of total 2HG (our assay measures both L-2-HG and D-2-HG) in our stable lines using MRM-MS. Our stable lines expressing IDH1-R132H-V5 showed low-moderate levels of 2HG in early passages and stabilized after passage #8, after which point they expressed approximately >15 fold 2HG compared to a stable line expressing IDH1-V5 (Supplementary Figure S3A). Subsequent experiments detecting 2HG were thus performed with cells after passage #8.

Next, we used MRM-MS to measure the levels of 2HG and 2KG in lines stably transfected with wild type and mutant expressing IDH and CIC constructs. Comparison of cells expressing mutant IDH1-R132H-V5 to cells expressing wild type IDH1 showed significant increases in 2HG (p<0.0001; Figure 5B). There were no significant differences in total 2HG levels between cells expressing wild type IDH1 alone or cells co-expressing wild type IDH1 with wild type F-CIC, F-CIC-R1515H, or F-CIC-R201W (Figure 5C). Cells expressing IDH1-R132H-V5 alone and cells co-expressing IDH1-R132H-V5/F-CIC, IDH1-R132H-V5/F-CIC-201W, or IDH1-R132H-V5/F-CIC-R1515H showed significant increases in 2HG levels (p<0.0001; p=0.0032, p<0.0001, respectively; Figure 5C). However, compared to cells co-expressing IDH1-R132H-V5/F-CIC, cells co-expressing IDH1-R132H-V5/F-CIC-R1515H further showed a significant increase in 2HG levels (p<0.0001; Figure 5C). In the IDH1-R132H-V5 background, the most significant increase in 2KG levels were observed in cells co-expressing F-CIC-R201W (p<0.0001; Figure 5C).

To assess whether our observations were specific to our HEK-derived lines, we derived stable HOG cell lines that ectopically expressed wild type CIC-S or mutant CIC-S (R1515H or R201W), in combination with wild type IDH1 or mutant IDH1-R132H. The HOG cell line was derived from a human glioma and expresses oligodendrocyte-specific proteins [34]. This cell line was previously determined not to harbour 1p19q co-deletions or mutations in the *IDH1* or *IDH2* genes [35, 36]. We determined 2HG levels in our stably transfected HOG cell lines using an enzymatic assay that is based on the detection of stoichiometrically generated NADH that was generated during the conversion of 2HG to 2KG in the presence of the enzyme (D)-2-hydroxyglutarate dehydrogenase (HGDH) and nicotinamide adenine dinucleotide (NAD^+^) [37]. In agreement with our HEK stable cell lines, comparison of cells co-expressing IDH1-R132H-V5 and F-CIC to cells co-expressing IDH1-R132H-V5 and F-CIC-R1515H showed a striking increase in 2HG levels (p<0.014; Figure 5D). Similar to our HEK cells, comparison of cells co-expressing IDH1-R132H-V5 and F-CIC to cells co-expressing IDH1-R132H-V5 and F-CIC-R201W did not show an increase in 2HG levels (p=0.79; Figure 5D). In contrast to the HEK-derived stable line, the HOG stable line expressing IDH1-R132H-V5 alone produced high levels of 2HG. However, co-expression of wild type F-CIC in combination with mutant IDH1-R132H significantly reduced the 2HG levels (p<0.003), indicating that CIC expression can modulate 2HG production. Taken together, our results indicate that expression of mutant CIC-R1515H, but not wild type CIC or CIC-R201W, can cooperate with mutant IDH1-R132H to increase cellular levels of 2HG (Figure 5E).

Examining the expression levels of F-CIC, F-CIC-R1515H, and F-CIC-R201W over passages (passage #2-15) in HEK-derived stable cells lines showed gradually decreasing levels of F-CIC, but both mutant F-CIC-R1515H and F-CIC-R201W proteins were stable (Supplementary Figure S3B). Although all the HEK-derived stable cell lines expressed similar levels of IDH1-R132H in early passages (passage #2-4), the stable line expressing mutant F-CIC-R1515H showed the highest level of IDH1-R132H-V5 protein in late passages (Supplementary Figure S3C). Similarly, the HOG-derived stable cell line expressing mutant F-CIC-R1515H showed the highest level of IDH1-R132H-V5 protein in later passages (Supplementary Figure S3D), which may explain the significant increase in 2HG levels in these lines.

### CIC interacts with metabolism and mitochondrial related proteins

To further shed insight into CIC function, we sought to identify CIC interacting proteins in the cytoplasm. We performed three co-immunoprecipitations (Co-IP) of cytoplasmic fractions using an anti-CIC antibody and rabbit IgG IPs as controls. We also transiently expressed CIC-S proteins with N-terminal FLAG, Myc or GFP tags in HEK cells, extracted the cytoplasmic fractions and performed IPs using anti-FLAG, anti-Myc or anti-GFP antibodies. Cells transfected with empty FLAG, Myc or GFP vectors served as controls. Isolated protein complexes were characterized using liquid chromatography and tandem mass spectrometry (LC-MS/MS). Consistent with a previous report showing interaction of CIC with Ataxin family members [10], we detected Ataxin2L in our IP experiments (Table 1). We also identified ACLY, a metabolism-related protein and 11 mitochondrial proteins as high-confidence candidate interaction partners of CIC in our LC-MS/MS experiments (Table 1).

**Table 1:** The candidate mitochondrial and metabolism related interaction partners of CIC Columns 1 and 2 indicate UniProt protein name and accession number. Column 3 indicates the gene name, synonym and function. A total of six immunoprecipitation experiments (IP), 4 IP experiments with an endogenous antibody (E-1, E-2, E-3, W-1), and 3 experiments with a Myc (M-1) or GFP (G-1) or FLAG (F-1) antibody of N-terminus CIC fusion proteins (MYC-CIC, GFP-CIC and FLAG-CIC, respectively) were conducted in HEK cells. Candidate proteins shown here were those observed in at least two independent IP experiments and not observed in the control IPs (4 IgG, and one each of FLAG, MYC or GFP empty vector) that were mitochondrial, or had metabolism related functions. Column 4 indicates the IP method for each candidate interaction partner. Column 5 indicates the Mascot score observed for each protein. Column 6 indicates the number of discrete (unique) peptides observed in the IP experiments.

UniProt			Protein identification information		
Name (Human)	Acession	Description; Gene name; Synonym (Function)	Type of IP	Mascot score	Discrete peptides
**metabolism:**					
ACLY	P53396	ATP-citrate synthase; ACLY;	E-1	135	3
		(ATP citrate-lyase)	E-2	388	9
			E-3	224	7
**Mitochondrial**					
ADT2	P05141	ADP/ATP translocase 2; SLC25A5; ANT2	E-2	104	2
		(exchange of cytoplasmic ADP with mitochondrial ATP)	G-1	392	8
ATD3A	Q9NVI7	ATPase family AAA domain-containing protein 3A; ATAD3A	E-2	349	8
		(mitochondrial network organization, mitochondrial DNA replication)	E-3	197	5
ATPA	P25705	ATP synthase subunit alpha, mitochondrial: ATP5A1; ATP5A	M-1	104	3
		(alpha subunit of F1-ATPase catalytic core)	G-1	96	3
ATPB	P06576	ATP synthase subunit beta, mitochondrial; ATP5B;	E-2	105	2
		(beta subunit of F1-ATPase catalytic core)	G-1	87	2
DHX30	Q7L2E3	Putative ATP-dependent RNA helicase DHX30; DHX30; DDX30	E-1	142	5
		(Associates with mitochondrial DNA)	E-2	64	2
			E-3	93	3
			M-1	584	12
DNM1L	O00429	Dynamin-1-like protein; DNM1L; DLP1	E-1	380	8
		(mitochondrial and peroxisomal division)	E-2	110	3
			W-1	374	10
EFTU	P49411	Elongation factor Tu, mitochondrial; TUFM;	E-2	184	3
		(mitochondrial protein sysnthesis)	G-1	206	4
GRP75	P38646	Stress-70 protein, mitochondrial; HSPA9;	F-1	1214	22
		(chaperone)	G-1	430	8
HMMR	O75330	Hyaluronan mediated motility receptor; HMMR; RHAMM	E-1	38	1
		(cell motility)	E-2	904	20
			W-1	151	4
MMAB	Q96EY8	Cob(I)yrinic acid a,c-diamide adenosyltransferase, mitochondrial; MMAB;	E-2	470	7
		(adenosylcobalamin biosynthesis)	E-3	200	5
PTCD3	Q96EY7	Pentatricopeptide repeat-containing protein 3, mitochondrial; PTCD3; MRPS39	E-1	291	6
		(Mitochondrial RNA-binding protein)	E-2	64	1
			M-1	41	1
**Other**					
ATX2L	Q8WWM7	Membrane; Peripheral membrane protein	E-1	754.9	17
			E-2	1316	26
			E-3	1223.5	26

**Figure 6:**
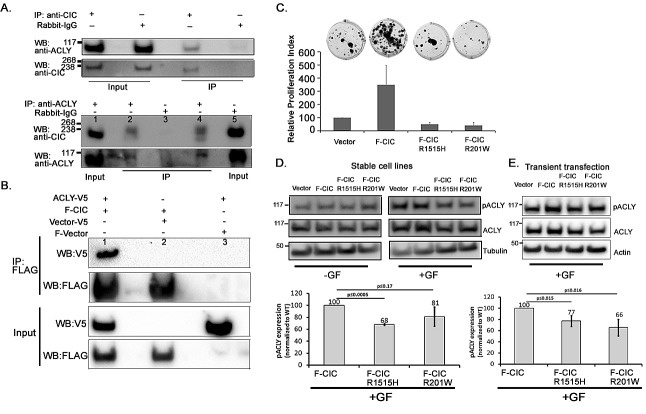
pACLY levels were low in cells expressing mutant CIC proteins A. Immunoprecipitations with an anti-CIC antibody or rabbit IgG using cytosolic (nucleus-free and mitochondria-free) HEK cell fractions were performed and ACLY was detected using an anti-ACLY antibody. A reciprocal immunoprecipitation using an anti-ACLY antibody or rabbit-IgG and the cytosolic fraction of HEK cells was also performed and CIC-S was detected using an anti-CIC antibody and western blot analyses. Lanes 1 and 5 are input lysates for IP with ACLY antibody and IgG respectively. Two aliquots of IP with anti-ACLY were loaded onto Lanes 2 and 4. A control IP with IgG is shown in lane 3. The figures are representative of three experimental replicates. B. FLAG-tagged CIC (bait protein) and V5 tagged ACLY (prey) were co-expressed in HEK293 cells. Co-immunoprecipitation was carried out using anti-FLAG antibody. CIC and ACLY were detected using anti-FLAG and anti-V5 antibodies respectively and western blot analysis. ACLY was detected only in the presence of FLAG-CIC (lane1) and although it was expressed, not detected in the absence of the FLAG-CIC (lane 3), thus confirming that CIC interacts with ACLY in these cells. C. Stable cell lines expressing vector, F-CIC, F-CIC-R1515H or F-CIC-R201W were exposed to serum free media containing growth factors. Proliferating cell colonies were observed two weeks after culturing using crystal violet stain. To generate proliferation indices, crystal violet stain retained in the clones was re-suspended in 10% acetic acid and measured at A_590_. Proliferation indices were calculated relative to cells expressing vector only (endogenous CIC). Standard deviation (SD) values shown are from 3 independent experiments. D. Stable cell lines expressing F-CIC or F-CIC-R1515H or F-CIC-R201W were exposed to serum free media with or without growth factors (+GF or –GF respectively) for 6 hrs. Levels of total ACLY and phosphorylated ACLY (pACLY) were determined using western blot analysis. Densitometry quantitations of pACLY/tubulin for stable cell lines expressing F-CIC or F-CIC-R1515H or F-CIC-R201W with growth factors relative to F-CIC expression are shown. SDs were calculated from three independent experiments. P-values shown were calculated using Student's t-test. E. HEK293 cells were transiently transfected with vector, F-CIC, F-CIC-R1515H or F-CIC-R201W. Cells were exposed to serum free media with growth factors (+GF) for 48 hrs after transfections. Levels of total ACLY and pACLY were determined using western blot analysis. Densitometry quantitations of pACLY/tubulin for stable cell lines expressing F-CIC or F-CIC-R1515H or F-CIC-R201W relative to F-CIC expression are shown. SDs were calculated from three independent experiments. P-values shown were calculated using Student's t-test.

Since ACLY is involved in cytosolic citrate metabolism and alterations in citrate metabolism are important in malignancies [24], we chose to further study the ACLY/CIC interaction. Because the ACLY/CIC interaction was observed in endogenous IP experiments (Table 1, Figure 6A), we confirmed the interaction using a reciprocal IP strategy. We used an anti-ACLY antibody and western blots to detect CIC-S in the IP derived from a HEK cytoplasmic fraction (Figure 6A). We also co-expressed F-CIC and ACLY fused to a C-terminal V5 epitope (ACLY-V5) in HEK293 cells. CIC immunoprecipitation using anti-FLAG antibody also captured ACLY; however, IP with anti-FLAG in the absence of F-CIC failed to immunoprecipitate ACLY (Figure 6B), compatible with the notion that CIC-S forms complexes with ACLY.

### Mutations in CIC lead to reduced pACLY

Phosphorylation of ACLY, including at Serine 454 (hereafter referred to as pACLY), contributes to increased ACLY stability and activity [38-40]. ACLY converts cytosolic citrate to acetyl CoA, which promotes cancer cell proliferation [40, 41]. IDH1 converts cytosolic isocitrate, derived from citrate, into alpha-ketoglutarate (2KG). Mutant IDH1-R132H further diverts this pathway towards the production of the oncometabolite 2HG (Figure 5D). These two pathways may thus compete for citrate. Having observed that CIC interacts with ACLY (Table 1, Figure 6A), we sought to determine the levels of ACLY or pACLY in cells expressing wild type or mutant CIC. The HEK-derived stable cell lines grown in DMEM+10%FBS did not show differences in growth or morphology. In contrast, when grown in serum free media with growth factors (ODG culturing media; see Methods), cells expressing mutant CIC formed reduced numbers of clones compared to cells expressing wild type CIC (Figure 6B). We measured the levels of total ACLY and pACLY after exposing our HEK stable cell lines to serum free media with or without growth factors for 6 hrs. Compared to cells expressing wild type CIC, we saw pACLY reductions in cells expressing either of the mutant CIC protein constructs but the most extreme reduction of pACLY was observed in cells ectopically expressing F-CIC-R1515H (p<0.0005) (Figure 6C). To confirm this observation, we transiently transfected wild type and mutant CIC proteins and measured the levels of ACLY and pACLY. Similar to the stable cell lines, cells transiently transfected with both F-CIC-R1515H and F-CIC-R201W showed reductions in pACLY (p<0.015 and p<0.016 respectively) compared to the wild type CIC (Figure 6E) supporting the notion that CIC mutations affect pACLY levels.

**Figure 7:**
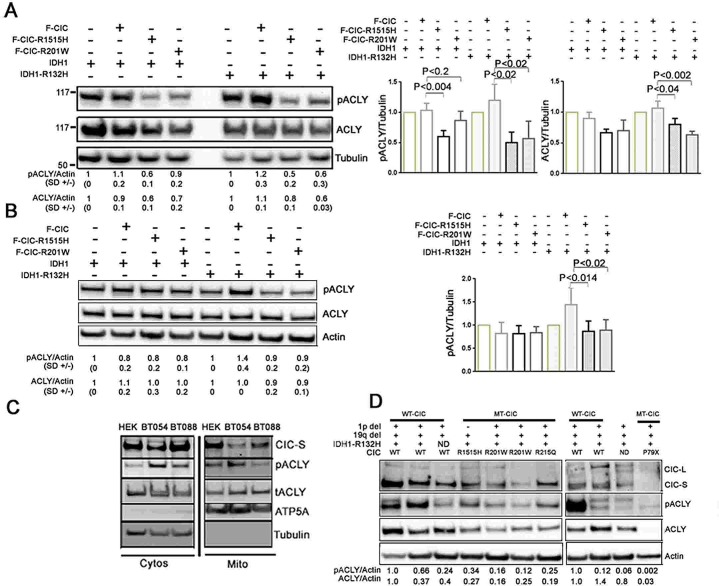
Cells expressing mutantCIC proteins exhibit reductions in pACLY A. Western blot analyses were used to detect total ACLY and pACLY in HEK-derived cells grown for 48hrs in serum free culturing conditions with 0.5% FBS and growth factors. Densitometry quantitations of pACLY/tubulin and ACLY/tubulin levels with standard deviations (SD) are shown below the western blots. Relative protein levels of pACLY and ACLY in cells expressing mutant CIC proteins (F-CIC-R1515H, or F-CIC-R201W) were derived relative to control cells expressing vector only. As shown in the bar graph, independent of IDH mutational status, CIC-R1515H mutation resulted in significant reduction in pACLY (p<0.01). The p-value was calculated from three independent experiments using one-way ANOVA analysis. B. Total ACLY and pACLY were also detected in independent HOG-derived stable cell lines grown for 72hrs in serum free culturing conditions with 0.5% FBS and growth factors. Similar to HEK-derived stable cells, independent of IDH mutational status, the highest level of reduction in pACLY was observed in cells expressing mutant CIC (-R1515H). C. Cytosolic and mitochondrial fractions were isolated from ODG cells BT054 (IDH1^+/R132H^; CIC^+/−^) and BT088 (IDH1^+^; CIC^R1515H/−^) and total ACLY and pACLY were detected in both cytosolic (Cytos) and Mitochondrial (Mito) fractions. The images shown are representative of 3 independent experiments. D. The levels of ACLY and pACLY in 1p19q co-deleted ODG tumours with known CIC and IDH1 status (n=10) were determined using western blot analysis. Densitometry quantitations of pACLY/tubulin and ACLY/tubulin levels were determined and are shown below the western blots. Tumours with wild type CIC (n=5) showed elevated levels of either ACLY or pACLY compared to the tumours with mutant CIC proteins (n=5). The most striking reduction in ACLY and pACLY was observed in a tumour sample with a truncated CIC protein (P79X).

We next investigated whether IDH1 and CIC mutations could affect ACLY or pACLY levels. We used our HEK and HOG-derived stable cell lines expressing mutant CIC in wild type or mutant IDH1 backgrounds. In HEK stable lines, we saw significant differences in the levels of pACLY in cells expressing F-CIC-R1515H compared to F-CIC on both IDH1-V5 (p<0.004) and IDH1-R132H-V5 (p<0.02) backgrounds (Figure 7A). Cells expressing F-CIC-R201W compared to F-CIC also showed significant differences in the levels of pACLY on IDH1-R132H-V5 backgrounds (p<0.02; Figure 7A). We saw significant differences in the levels of total ACLY in cells expressing either F-CIC-R1515H or F-CIC-R201W compared to F-CIC in IDH1-R132H-V5 backgrounds (p<0.02 and p<0.001 respectively; Figure 7A). In HOG stable lines, we also saw reduced levels of pACLY in cells co-expressing mutant IDH1-R132H-V5 and mutant F-CIC-R1515H or F-CIC–R201W compared to wild type F-CIC (p<0.014 and p<0.02 respectively; Figure 7B).

We next assessed pACLY levels in ODG cell lines with wild type CIC (BT054; IDH1^+/R132H^/CIC^+/−^) and mutant CIC-R1515H (BT088; IDH1^+/+^/CIC^R1515H/−^). In agreement with the results we obtained using our stably transfected cell lines, reduced pACLY levels were observed in BT088 cells compared to BT054 cells, both in the cytoplasmic and mitochondrial fractions (Figure 7C). We next measured ACLY and pACLY levels in 1p19q co-deleted oligodendroglioma patient samples with known CIC and IDH1 status. In agreement with our *in vitro* findings, tumours with wild type CIC (n=5) showed elevated levels of either ACLY or pACLY compared to tumours with mutant CIC proteins (n=5; Figure 7D). Strikingly, a tumour sample with a truncated CIC protein (P79X) showed dramatic reductions in ACLY and pACLY. We interpret these findings to indicate that wild type CIC forms protein complexes with ACLY, and CIC mutants act to reduce the levels of pACLY and/or ACLY. As depicted in Figure 5C and D, reduction in ACLY/pACLY levels in our cells and in 1p19q co-deleted tumours expressing mutant CIC is consistent with the notion that mutations in CIC facilitate the diversion of citrate towards the production of 2HG.

### Mutations in CIC and IDH1 cooperate to reduce proliferation and clonogenicity

**Figure 8:**
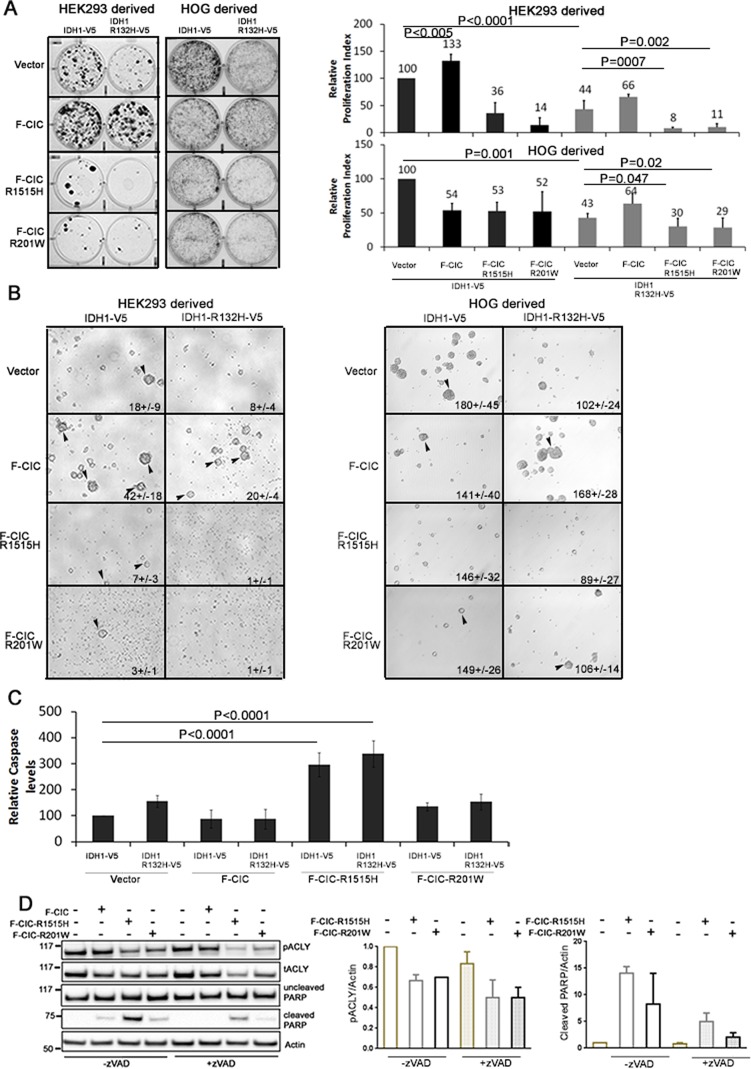
CIC and IDH1 coordinately regulate cell clonogenicity and mutations in CIC regulate cell death by reducing pACLY levels *in vitro* A. Clonogenic assays of HOG and HEK-derived stable cell lines were performed in serum free media with low FBS and growth factors. Proliferating cell colonies were visualized using crystal violet stain one week (HOG) or two weeks (HEK) after culturing. Proliferation indices were calculated relative to cells expressing IDH1-V5 and vector only (endogenous CIC). Error bars represent the SD from 3 independent experiments. Multiple comparisons using mean values were performed using one-way ANOVA. B. Stable cell lines were cultured under oligodendroglioma cell growing conditions without FBS to form spheres. Spheres were viewed under an inverted light microscope. Images were taken 1 week after plating. Compared to cells expressing wild type IDH1 only or wild type IDH1 and CIC, sphere formation was reduced in cells expressing mutant CIC and mutant IDH1. Sphere counts (+/− standard deviation) represent results obtained from 3 independent experiments. C. Stable cell lines were grown in serum free media with 0.5% FBS and growth factors for 72hrs and caspase 3 and 7 expression was determined using the Caspase-Glo® 3/7 assay. Caspase levels were calculated relative to cells expressing IDH1-V5 and vector only (endogenous CIC). Cells expressing F-CIC-1515H showed the highest levels of caspase activity (p<0.0001). Error bars represent the SD from 3 independent experiments. D. Stable lines expressing mutant IDH1-R132H-V5 combined with wild type F-CIC or mutant F-CIC-R1515H or F-CIC-R201W were cultured for 48hrs with or without 10μM zVAD (a pan caspase inhibitor) to determine whether reduction in pACLY led to cell death. Levels of total ACLY, pACLY and cleaved PARP were detected using western blots. zVAD treatment significantly reduced cleaved PARP in cells expressing mutant CIC. Western blots are representative of 3 biological replicates and the bar graphs shown are densitometry quantitations from 3 biological replicates.

Since pACLY levels were reduced and 2HG levels were increased in cells expressing CIC-R1515H and IDH1-R132H, we predicted that mutant CIC, in cooperation with mutant IDH1, may alter cell proliferation, survival or death. To compare the proliferation of cells carrying F-CIC-R1515H or F-CIC-R201W and IDH1-R132H-V5 to cells expressing wild type F-CIC with IDH1-V5, we performed clonogenic assays combined with crystal violet proliferative assays (hereafter referred to as crystal violet assays) under serum free conditions supplemented with 0.5% Fetal Bovine serum (FBS) and growth factors. Without 0.5% FBS, both HEK and HOG cells do not adhere and form spheres; however, with 0.5% FBS, HEK and HOG cells can adhere and proliferate. In HEK stable cells, ectopic co-expression of F-CIC with IDH1-V5 showed a 33% relative increase in proliferation compared to cells ectopically expressing IDH1-V5 alone (p<0.005; Figure 8A). Expression of IDH1-R132H-V5 alone decreased the relative proliferation of cells (44%+/−12; p<0.001) compared to IDH1-V5. Ectopic co-expression of F-CIC with IDH1-R132H-V5 partially rescued this low proliferative phenotype (Figure 8A). These findings suggest that CIC and IDH1 can cooperate to influence cell proliferation *in vitro*. Cells expressing mutant CIC proteins R1515H or R201W in a wild type IDH1 background showed significant decreases in relative cell proliferation compared to F-CIC with IDH1-V5 co-expression (reduced by 97% and 119% respectively; p<0.0001 for both). Compared to cells expressing IDH1-R132H-V5 or F-CIC-1515H alone, cells co-expressing IDH1-R132H-V5/F-CIC-1515H showed further reductions (36% and 28% respectively) in relative cell proliferation (Figure 8A). Similarly, in HOG-derived stable lines, expression of IDH1-R132H-V5 alone decreased the relative proliferation of cells (43+/−7%; p<0.001) compared to IDH1-V5; ectopic co-expression of F-CIC with IDH1-R132H-V5 partially rescued the low proliferative phenotype (Figure 8A). Compared to cells expressing IDH1-R132H-V5, cells co-expressing IDH1-R132H-V5 and F-CIC-1515H or IDH1-R132H-V5 and F-CIC-201W showed further reductions (13 and 14% respectively) in relative cell proliferation (Figure 8A). Similar observations were also found in media without FBS in both HEK293 and HOG-derived stable cell lines (Figure 8B). Cells co-expressing wild type CIC and mutant IDH1 formed numerous spheres that were also larger in size compared to cells expressing mutant IDH1 and either mutant CIC (-R1515H/-R201W) proteins (Figure 8B). These findings are in agreement with the notion that mutations in IDH1 and CIC are co-operatively regulating cell clonogenicity. Although cells expressing IDH1-R132H-V5 and F-CIC-R1515H or IDH1-R132H-V5 and F-CIC-R201W exhibited dead cells after one week, we did observe survival of a small fraction of cells that continued to thrive.

In stable HEK cells expressing mutant F-CIC-R1515H, we observed cells displaying anoikis after 48 hours in culture. To assess whether these cells were undergoing caspase dependent cell death, we measured the activity of caspases 3/7 and the levels of cleaved PARP (product of caspases 3 and 7 activity) in our CIC wild type/mutant HEK-derived stable lines in combination with IDH1-R132H-V5. Cells expressing F-CIC-R1515H in either the IDH1-V5 or IDH1-R132H-V5 background showed significant increases (Figure 8C; p<0.0001) in caspase activity and yielded higher levels of cleaved PARP compared to vector only, wild type CIC and CIC-R201W (Figure 8D). Reduction in ACLY and pACLY elicited cell death in non-small cell lung cancer cells [42] and pancreatic beta cells [43], and so we sought to confirm this in our model system. To inhibit caspase activity, we treated cells with a pan caspase inhibitor Z-VAD-FMK (zVAD). After 48 hours in culture, cells displaying anoikis were not observed in zVAD treated cells. Western blot analysis showed dramatic reduction of cleaved PARP in F-CIC-R1515H zVAD treated cells (Figure 8D) compared to untreated cells and showed that pACLY levels were lower in these cells. These findings are compatible with the notion that reductions in pACLY led to anoikis and cell death. Increased caspase activity and reduced clonogenicity in cell lines expressing mutant IDH and the R1515H mutant CIC *in vitro* appears to mimic the slow growing nature of ODGs and may explain why it is difficult to grow ODGs with IDH1 and CIC mutations *in vitro* [30, 44, 45]. It is noteworthy that to date, no 1p19q co-deleted ODG cell lines with both IDH1 and CIC point mutations are available, despite 70% of ODGs carrying mutations in both genes.

## DISCUSSION

Although CIC has been implicated in human diseases including cancers, the biology of CIC in mammalian cells remains poorly understood. Using immunoblotting and *MRM mass spectrometry assays,* we have confirmed the presence of two forms of endogenous CIC in HEK, oligodendroglioma (ODG) cells and tumour tissues (Figure 1, Figure 7). We further determined the subcellular localization of CIC-S (cytoplasm) and CIC-L (nucleus) in human cells. Localization of these isoforms to different compartments may indicate that these isoforms have distinct functions.

CIC was reported to be a transcriptional repressor in *Drosophila* nucleii and in mammalian cells [16, 17]. As far as we are aware, ours is the first study to report the localization of endogenous CIC-S and its proximity to mitochondria, the first study to report CIC-protein interactions and the first study to link CIC and mutations therein to roles in citrate metabolism. Cytosolic citrate is converted into 2KG by IDH1 and IDH1-R132H mediates the conversion of 2KG into 2HG, which accumulates at high levels in cells. ACLY on the other hand, converts cytosolic citrate into Acetyl-CoA that is essential for *de novo* lipid synthesis. In this paper, we established a possible mechanism of cooperative regulation of cytosolic citrate by mutant IDH1 and CIC.

Since IDH1 is the most frequently mutated gene in 1p19q co-deleted ODG and CIC is mutated in ~70% of oligodendrogliomas, we studied the effects of expressing wild type and mutant CIC in combination with IDH1-R132H on cell proliferation, cell survival and cell death using ODG cell culturing conditions. We for the first time showed a cooperative regulation of 2HG in mammalian cells expressing both mutant IDH1-R132H-V5 and the R1515H mutant CIC. Since high levels of 2HG can be toxic to cells [26], the cooperative increase in 2HG in the mutant cells expressing both IDH1-R132H-V5 and F-CIC-R1515H may explain at least in part the cooperative reduction in clonogenicity in these stable cell lines under ODG culturing conditions. Among the somatic mutations observed in CIC in gliomas, 41% and 7% of all missense mutations were observed in the HMG binding domain and protein binding domain, respectively [2, 5, 7, 8]. Since HEK293 cells co-expressing IDH1-R132H-V5 with F-CIC or F-CIC-R201W produce less 2HG compared to the cells co-expressing IDH1-R132H-V5 and F-CIC-R1515H, it is tempting to speculate that in gliomas, missense mutations in the HMG domain alter its function to produce a 2HG level for tumour cells that balances cell survival and increased tumorogenicity, and for this reason such mutations are observed more frequently than missense mutations in the protein binding domains. In contrast to HEK293 cells, HOG-derived cells expressing IDH1-R132H-V5 alone also showed elevated levels of 2HG and co-expression of IDH1-R132H-V5/ F-CIC significantly reduced the 2HG levels. Even so, co-expression of IDH1-R132H-V5 and F-CIC-R1515H resulted in increased 2HG compared to HOG cells co-expressing either wild type F-CIC or mutant F-CIC-R201W, consistent with the HEK results. HOG cells differ from HEK cells in several ways, and so this level of concordance is likely reflective of underlying biology, not technical artifact.

The levels of ACLY/pACLY modulate tumourigenicity in gliomas [20]. Increased abundance of ACLY and pACLY was also associated with proliferation in other types of aggressive cancers [40, 42, 46-48]. In glioma cells, increased pACLY was observed in pseudopodia of U87 glioblastoma cells and was associated with increased clonogenicity and cell migration [20]. In other studies, inhibition or reduction of ACLY suppressed cell proliferation [38, 49, 50]. Consistent with these findings, we assigned a novel function to mutant CIC-S in reducing ACLY/pACLY levels in our cell lines and in human tumour samples and correlated this reduction with reduced clonogenicity *in vitro*.

Whether mutations in IDH1 promote or inhibit cell proliferation and survival depends on the tumor site. While mutations in IDH genes in Acute Myeloid Leukemia have been associated with poor prognosis, the same mutations are considered as markers of improved prognosis in gliomas, indicating interactions with other proteins or the tumour environment may ultimately influence the role of IDH in tumour pathogenesis. While several studies of glioma patient samples [51, 52] found no correlation between IDH1 mutations and tumour invasiveness, another study [53] in a fibrosarcoma cell line with endogenous IDH1-R132C showed a requirement for mutant IDH1 protein for cell survival. Our study is in agreement with studies [54, 55] in glioma cell lines stably transfected with IDH1-R132H-V5, where reduced proliferation and cell migration *in vitro* and *in vivo* were reported.

Our study showed for the first time a cooperative reduction in clonogenicity in cells co-expressing IDH1-R132H and mutant CIC proteins. A cooperative phenotype of lower proliferation driven by co-expression of mutant IDH1 and mutant CIC proteins in HEK293 and HOG stable cell lines is at least in part in agreement with several important observations in gliomas: (a) Glioma patients with IDH1-R132H or IDH2-R172K had slower tumour progression, longer survival and better response to radiation or Temozolomide treatment [55, 56]; (b) Mutations in CIC were almost always observed in 1p19q co-deleted ODG and patients with this type of ODG had slower tumour growth and longer overall survival compared to ODG without co-deletion [2, 4]; (c) Transgenic mouse models expressing IDH1-R132H in brain exhibit hemorrhage, reduced proliferation and increased apoptosis and failed to spontaneously develop brain tumors [57]; (d) Attempts to culture ODG cells *in vitro* failed to grow ODG lines that harbor both IDH1 and CIC mutations [44, 58].

Although the reduced clonogenicity and cell proliferation of IDH1 and CIC mutations alone or in combination are in agreement with the slow growing nature of ODG with 1p19q co-deletions, the selective advantage that slow growth confers on malignant cells is unclear. One explanation may be that other oncogenic proteins or pathways may drive or be permissive for tumour progression of ODGs initially. In line with this hypothesis, a recent study by Killela and colleagues [9] identified recurrent mutations in the C228T or C250T positions of *TERT* promoters in ODGs with 1p19q deletions and suggested that these mutations may promote a self-renewal capability in these tumours. It is possible that the mutations in IDH1 and CIC may in fact be moderately deleterious and reduce clonogenicity but may provide other timely survival advantages under certain conditions during oligodendroglioma disease progression.

## MATERIALS AND METHODS

### Cell culture, cell lysate preparations and western blot analysis

HEK293A, MDA-MB-231, HOG, and HeLa cell lines were cultured in DMEM (Invitrogen) supplemented with 10% (v/v) heat inactivated FBS (Invitrogen). C643 and H522 cell lines were cultured in RPMI (Invitrogen) supplemented with 10% (v/v) heat inactivated FBS. Stable cell lines derived from HEK293A or HOG were cultured in DMEM+10% FBS with selection (G418 and zeocin). Unless otherwise indicated, experiments with stable cell lines were always performed between passages 8-14. Cells were cultured without selection for at least two passages before experiments. Cell culturing was performed in a *humidified,* 37°C, *5*% *CO2* incubator. Cell lysate preparations, cell fractionations and western blot analysis were performed using standard protocols and are described in detail in the Supplementary data S1.

### Immunoprecipitation, mass spectrometry and identification of candidate interaction partners

For CIC immunoprecipitation, myc-CIC-S or rabbit/mouse IgG (control) antibody-bound beads were prepared by incubating anti-CIC antibody, anti-myc antibody, normal rabbit-IgG, or mouse-IgG with protein G agarose beads (Roche) in IP buffer (20mM Tris-HCl pH 7.5, 150mM NaCl, 1mM EDTA with 1X complete protease inhibitor from Roche) at 4°C overnight. Cell lysates were first pre-cleared with Sepharose 4B beads (Sigma) at 4°C for 1 hour and the lysates were immunoprecipitated with the prepared protein-G beads at 4ºC for 4 hours. The captured proteins and protein complexes were released by boiling the beads in 2X LDS Sample Buffer (Invitrogen) at 95^o^C for 5 minutes or 2X SDS sample buffer (117mM Tris-Cl pH6.8, 4% SDS, 8% glycerol, 0.01% bromophenol blue, 200mM DTT) at 98^o^C for 10 minutes (see supplementary for details). For immunoprecipitations using FLAG epitopes, complexes in the pre-cleared lysate were captured by anti-FLAG M2 Affinity agarose (Sigma) for 2 hours at 4°C. Captured proteins and complexes were eluted by two rounds of FLAG-peptide competition (400ug/mL FLAG peptide, 50mM ammonium bicarbonate) for 30 minutes at 4°C.

### Serum free cell culturing

Cells were first plated in DMEM+10% FBS. The following day, media were replaced with Neurocult proliferation media supplemented with cytokines (Stem Cell Technologies) and growth factors EGF, FGF (20μg/ml each; Peprotech) and Heparin (0.1%; Stem Cell Technologies) and cells were grown for the time periods indicated. For caspase inhibition assays, cells were grown with or without the pan caspase inhibitor zVAD-FMK (BD Biosciences)

### Clonogenic and crystal violet cell proliferation assays

Stable cell lines were plated at 1×10^4^ cells per well in a 6-well plate. Cells were grown in ODG media for up to 2weeks. Colonies were fixed with 4% paraformaldehyde and stained with 10% crystal violet. Images of colonies were captured using a Bio-Rad imaging system. Retained crystal violet stain was resolubilized in 1ml 10% acetic acid by rocking for 15 min. Readings were taken at A_590_ using a plate reader (VersaMAX Microplate Reader).

### Statistical analysis

For multiple comparisons of western blot results, 2HG/2KG levels and clonogenic crystal violet proliferation assays, one-way ANOVA with Dunnett's post-test were performed using Prism 6.0 Software (GraphPad Software Inc.).

For further descriptions of Materials and Methods, please see the Supplementary Materials and Methods.

## SUPPLEMENTARY MATERIAL FIGURES


