# Supplementary material for: Mutations in CIC and IDH1 cooperatively regulate 2-hydroxyglutarate levels and cell clonogenicity
Source: Oncotarget. 2014 Aug 27;5(17):7960–79. doi: 10.18632/oncotarget.2401 (PMC4202173; doi:10.18632/oncotarget.2401)
Supplement: Supplementary file 1 [file oncotarget-05-7960-s001.pdf]

Mutations in CIC and IDH1 cooperatively regulate 2-hydroxyglutarate levels and cell clonogenicity

Supplementary Material
Detailed Materials and Method
Antibodies and Reagents:
Normal rabbit-IgG and mouse-IgG were obtained from Santa-Cruz. Anti-CIC antibody A301-204A (Bethyl Laboratories) was used for immunofluorescence and western blot analysis. Anti-CIC antibody A301-203A (Bethyl Laboratories) was used for immunoprecipitations. The following primary antibodies were also used for immunofluoresce, western blot analysis or immunoprecipitations: anti-c-myc antibody (clone 9E10, Roche), anti-FLAG antibody (F3165, Sigma), anti-tubulin (sc-9104, Santa Cruz), anti-TATA binding protein (TBP) (ab818, Abcam), ACLY (15421-1-AP, Proteintech Group), pACLY (4331S, New England BioLabs (NEB)), anti-ATP5A antibody (ab14748, Abcam), anti-ATAD3 antibody (ab67992, Abcam), or anti-Drp-1 antibody (ab56788, Abcam), VDAC (ab14734, abcam), HMMR (3139-1, Epitomics), anti-V5 antibody (V8012, Sigma). Alexa Fluor® 488 Goat Anti-Rabbit IgG (Invitrogen) or Alexa Fluor® 546 Goat Anti-Mouse IgG (Invitrogen) were used as secondary antibodies for immunofluorescence. For Infra Red (IR) western blot analysis, anti-mouse IRDye 800Dx (610-132-121, Rockland) and anti-rabbit IRDye 700Dx (611-130-122, Rockland) were used as secondary antibodies. For chemiluminescent western blot analysis, Goat anti-mouse IgG HRP (SC-2005, Santa Cruz) and Goat anti-rabbit IgG HRP (SC-2004, Santa Cruz) were used.
Immunofluorescence assay:
Cells (1.47×10^4 per chamber) were cultured overnight on LabTekII® Chamber slides (Nunc) in DMEM (Invitrogen) supplemented with 10% FBS (Invitrogen). Cells were fixed for 20 min in 2% (w/v) paraformaldehyde, and then for an additional 10 min in 4% (w/v) paraformaldehyde. Lymphoblastoid and oligodendroglioma cells (1.47×10^4) were attached to charged slides using a Shandon Cytospin®4 Centrifuge (Thermo Scientific), and then fixed for 20 min in 4% (w/v) paraformaldehyde, followed by two PBS washes. Cells were permeabilized using 0.2% Triton X-100 for 5 min and blocked for 1 h in 1% BSA before incubation with primary antibodies overnight at 4°C. Cells were then incubated in secondary antibodies conjugated to Alexa Fluor® 488 or Alexa Fluor® 546 for 2 hours at room temperature. Cells were incubated in PBS containing Draq5 (Biostatus Ltd) and 100ug/mL RNAseA (Clonetech) to visualize nuclei. Slides were mounted in SlowFade® without DAPI (Invitrogen). Images were acquired on a Nikon confocal microscope. Mitochondria were visualized either using Mitotracker red staining or with anti-ATP5A (ATP-synthase, mitochondrial protein) antibody. For MitotrackerRed (Invitrogen) staining cells were incubated in 500nM MitotrackerRed for 30 min at room temperature prior to fixing.
Proteinase K protection assay:
Lysed mitochondria (control) were prepared by incubating mitochondrial fractions in 50mM Tris, (pH7.5) for 30min (swelling buffer) on ice with vigorous vortexing every 10min. Both intact (0-15µg/ml) and lysed (0-5 µg/ml) mitochondria were incubated in Proteinase K (Roche) on ice for 15 min. Proteinase K was inactivated by adding PMSF (Fluka) to a final concentration of 1mM. Intact mitochondria were spun down at 4°C,

12,000 rpm for 20 min while lysed samples were Trichloroacetic acid (TCA) precipitated. These were then resuspended in 25ul gel loading buffer (NuPAGE LDS Sample Buffer, Invitrogen) for Western analysis.

Carbonate assay:

Intact mitochondrial pellets were subject to alkaline extraction with carbonate buffer as described previously [1]. Briefly, mitochondria were resuspended and incubated in 1 ml of 100 mM sodium carbonate buffer at pH 11 for 30 min at 4°C. Following centrifugation at 16,000g, the pellet was resuspended in 1XNuPAGE LDS (Invitrogen) sample buffer. The supernatant was TCA precipitated and resuspended in LDS sample buffer.

V5-tagged IDH1^{WT} and IDH1^{R132H} Plasmid Construction:

IDH1^{WT} and IDH1^{R132H} coding regions were first synthesized using the standard SMART cDNA protocol (Clontech) and 50ng placenta RNA (wildtype IDH) or RNA isolated from oligodendroglioma tumour cells (IDH1-R132H). Reverse transcribed coding regions were cloned into the GATEWAY entry vector. For cDNA cloning, AGTCTGCAAGACTGGGAGGA forward and NNNNNNNNGGTCACACGCTCCAGGTTAT reverse primers were used. For GATEWAY cloning, forward primer GGGGACAAGTTTGTACAAAAAAGCAGGCTTCACCatgtccaaaaaaatcagtggc and reverse primers GGGGACCACTTTGTACAAGAAAGCTGGGTCaagtttggcctgagctagtttg (upper and lower case here indicates, GATEWAY and gene specific sequences) were used. Using Gateway technology (Invitrogen), each IDH1^{WT} and IDH1^{R132H} open reading frame was cloned into pcDNA-DEST40 vector (Invitrogen) for the expression of C-terminal V5 fusion proteins.

Wild type and mutant N-terminal FLAG-CIC-short form (FLAG-CIC-S) plasmid construction:

A tandem 3x FLAG sequence was PCR amplified from plasmid pAFW1111 (Drosophila Genomics Resources Centre, Indiana, USA) using KAPA HiFi DNA polymerase (KAPA Biosystems). 5’-ATCGAAGCTTACCATGGACTACAAAGACCATGACG-3’ (F=forward) and 5’-ATCGGGATCCCTTGTCATCGTCATCCTTGTA-3’ (R=reverse) primers were used generate tandem FLAG amplicons with HindIII and BamHI restriction sites. pcDNA™4/TO vector (Invitrogen) was modified by inserting the 3x FLAG sequence at HindIII/BamHI sites to create N-terminus 3xFLAG expression vector (pcDNA™4/TO/N-FLAG).

pcDNA5 FRT/TO GFP-CIC construct (wild type) was obtained from University of Dundee, Scotland [2] . Site-directed mutagenesis was performed to create mutant constructs pcDNA5 FRT/TO GFP-CIC_R1515H and pcDNA5 FRT/TO GFP-CIC_R201W using QuikChange II XL Kit (Agilent). Mutant constructs were sequence verified. Using 5’-ATCGGAATTCAAGATCTATGTATTCGGCCCACAGGCCC-3’ (F) and 5’-AAGGCCAGGTCGTGGTACTTCT-3’ (R) primers and **TaKaRa LA Taq (Clontech)**, the first 1496 bp of CIC-S (referred to as 5’ CIC-S), including a BglII restriction site just before the start codon, was amplified from pcDNA5 FRT/TO GFP-CIC. The 5’ CIC-S amplicon was sequence verified and restriction digestion was performed using BglII (NEB).

A 4436 bp region of CIC from pcDNA5 FRT/TO GFP-CIC was obtained using StuI (NEB) and NotI (NEB) enzymes (referred to as 3’ CIC). This 3’ CIC fragment was ligated to the 5’ CIC-S amplicon using Quick T4 DNA ligase (NEB) to yield full length

CIC-S. CIC-S was cloned into BamHI/NotI sites of pcDNA™4/TO/N-FLAG to create pcDNA4/TO/FLAG-CIC-S (referred to as FLAG-CIC-S) plasmid construct. pcDNA4/TO/FLAG-CIC_R1515H and pcDNA4/TO/FLAG-CIC_R201W were created as described for FLAG-CIC-S.

Wild type N-terminal FLAG-CIC-long form (FLAG-CIC-L) plasmid construction:
Total RNA was extracted from HEK293A cells using AllPrep DNA/RNA Mini Kit (Qiagen) as per the manufacturer's instructions. cDNA capturing novel CIC Exon 0 was reverse transcribed from total RNA using primers 5'- GTTGCACCACTTCCAATCTGG-3' and 5'- GGCCTCTGAGCTGGACTTCTTT-3' with AccuScript High-Fidelity 1st Strand cDNA Synthesis Kit, as per the manufacturer's recommendations. The first strand cDNA product was used as a template for PCR with primers 5'-ATCGAGATCTTGAGTTGCTCCGCGTAATCC-3' (F) and 5'- AAGGCCAGGTCGTGGTACTTCT-3' (R), using TaKaRa LA Taq DNA polymerase. The 3548bp product, designated 5' CIC-L, was restriction digested with BglII (NEB) and StuI (NEB). 5' CIC-L was ligated with 3' CIC to generate full length CIC-L, and then cloned to generate pcDNA4/TO/FLAG-CIC-L.
All constructs were verified using DNA sequencing. Large-scale plasmid preparations were made using Qiagen Plasmid Maxi Kit as per the manufacturer's recommendations.

Wild type N-terminal FLAG-CIC-long form (FLAG-CIC-L) plasmid construction:
Total RNA was extracted from HEK293A cells using the AllPrep DNA/RNA Mini Kit (Qiagen) as per the manufacturer's instructions. cDNA capturing novel CIC Exon 0 was reverse transcribed from total RNA using primers 5'- GTTGCACCACTTCCAATCTGG-3' and 5'- GGCCTCTGAGCTGGACTTCTTT-3' with the AccuScript High-Fidelity 1st Strand cDNA Synthesis Kit, as per the manufacturer's recommendations. The first strand cDNA product was used as a template for PCR with primers 5'-ATCGAGATCTTGAGTTGCTCCGCGTAATCC-3' (F) and 5'- AAGGCCAGGTCGTGGTACTTCT-3' (R), using TaKaRa LA Taq DNA polymerase. The 3548bp product, designated 5' CIC-L, was restriction digested with BglII (NEB) and StuI (NEB). 5' CIC-L was ligated to 3' CIC to generate full length CIC-L , which was cloned to generate pcDNA4/TO/FLAG-CIC-L.
All constructs were DNA sequence verified. Large-scale plasmid preparations were performed using Qiagen Plasmid Maxi Kit as per the manufacturer's instructions.

Establishing Vector only, F-CIC, F-CIC-R1515H and F-CIC-R201W stable cell lines.
HEK293A cells were grown to 70%-80% confluency in a 6-well plate in DMEM (Invitrogen) supplemented with 10% (v/v) heat inactivated FBS (Invitrogen). 3ug of each plasmid was used to transfect cells using TurboFect Transfection Reagent (Thermo Scientific). Cells were treated with 50ug/mL Zeocin (Invitrogen) for 12-14 days, by which time untransfected cells treated with Zeocin had died. Expression of F-CIC and mutant CIC (F-CIC-R1515H or F-CIC-R201W) were verified using western blot analysis.
For IP experiments, an expression construct containing myc-CIC-S was obtained from Dr Huda, Howard Hughes Medical Institute, Texas [3] and a stable cell line was generated.

Establishing vector only, F-CIC, F-CIC-R1515H and F-CIC-R201W stable cell lines in wild type IDH1 and mutant IDH1 (R132H) backgrounds.

To co-express wild type IDH1 and CIC or wild type IDH1 and mutant CIC or mutant IDH1 and mutant CIC, stable cell lines expressing vector only or wild type F-CIC or mutant F-CIC (R1515H or R201W) were used as parental cell lines and transfected with either wild type IDH1-V5 or mutant IDH1-R132H constructs. Stable transfectants were selected using 50ug/mL Zeocin (Invitrogen) and 1mg/mL Geneticin (Invitrogen). After 12-14 days, protein expression was verified by western blot using anti-CIC (Bethyl, A301-204A), anti-V5 (Sigma, V8012), and anti-IDH1 R132H antibodies (Dianova, DIA-H09).

To co-express wild type IDH1 and CIC or wild type IDH1 and mutant CIC or mutant IDH1 and mutant CIC in HOG lines, constructs were co-transfected and selected using 50ug/mL Zeocin (Invitrogen) and 1mg/mL Geneticin (Invitrogen).

Cell culturing of stable cell lines:

Cells stably expressing myc-CIC were cultured in DMEM supplemented with 10% FBS and Geneticin (0.5 ug/mL; Invitrogen). HEK293A stable cell lines expressing wildtype FLAG-CIC-L, FLAG-CIC-S and mutant FLAG-CIC-S (-R1515H or -R201W) were cultured in DMEM supplemented with 10% (v/v) heat inactivated FBS and 10 ug/mL Zeocin (Invitrogen). All HEK293A and HOG stable cell lines co-expressing wildtype or mutant CIC and wildtype or mutant IDH1 were cultured in DMEM supplemented with 10% (v/v) heat inactivated FBS, 10 ug/mL Zeocin, and 200ug/mL Geneticin.

Transient transfections:

HEK293 cells were plated at $5x10^6$cells/well in a 6 well plate. The following day, cells were transfected with vector, F-CIC, F-CIC-R1515H, or F-CIC-R201W. 6hrs after transfections, cells were exposed to serum free media with growth factors (+GF) for 48. Cells were harvested and analyzed using western blots.

siRNA transfections:

HEK293 cells were plated to 70-80% confluency and transfected with 75nM stealth siRNA (Life Technologies), siGenome (Thermoscientific) or ON-Targer siRNA (Thermoscientific) against CIC with their respective controls as per the manufacturer's protocols. CIC knock-downs were evaluated using anti-CIC antibody (A301-204A, Bethyl Laboratories).

Whole cell lysate extraction

Cells were dislodged from tissue culture plates by incubating with 1X citrate saline solution (135mM KCl, 12mM sodium citrate) at room temperature for 2-3min. Cells were collected in phosphate buffered saline (PBS) and pelleted by centrifugation. Cell pellets were flash frozen and stored at -80C until use. Cells were thawed on ice and received 3X packed-cell volumes of freshly prepared ice-cold lysis buffer (20mM Tris-HCl pH 7.5, 150mM NaCl, 1mM EDTA, 0.5% NP-40, 10mM Sodium orthovanadate, 50mM beta-glycerophosphate, 1X cOmplete protease inhibitor (Roche)). Cell pellets were homogenized by 25 passages through a 21-gauge needle then mixed for 30 minutes at 4ºC on an automatic rotator. The remaining cellular debris was pelleted by centrifugation at 13000 x g for 30 minutes.

Crude cytosolic and nuclear protein extraction

Harvested cell pellets were washed 2 times with PBS followed by 10 minute incubations in 5X packed-cell volume of ice-cold Buffer A (20mM NaCl, 20mM Tris-HCl pH 7.5, 1mM EDTA, 1X complete protease inhibitor) on ice. 0.01% v/v NP-40 was added immediately before centrifugation at 300 x g to remove intact crude nuclei.

Pelleted crude nuclei were treated with Buffer A two more times for 5 minutes on ice before nuclei were lysed using 3X packed-nuclei volume of ice-cold Buffer B (10mM Tris-HCl (pH 7.2), 150mM NaCl, 2mM MgCl2, 1mM EDTA, 1X Roche cOmplete protease inhibitor, 0.05% NP-40). The nuclei pellet was homogenized by passage through a 21-gauge needle. Cytosolic and nuclear protein extracts were centrifuged at 13 000 x g for 30 minutes to remove insoluble cellular material.

Crude Mitochondrial Fractionation and Protein Extraction

Harvested cell pellets were washed twice with cold PBS, then resuspended in 5X packed cell volume of ice-cold SEM buffer (10mM MOPS pH7, 320mM sucrose, 1mM EDTA, 1X cOmplete protease inhibitor cocktail (Roche)). The cell suspension was homogenized with 100 strokes of a chilled dounce tissue grinder. Intact crude nuclei were removed by centrifugation two times at 3000 rpm for 5 min at 4°C. The supernatant was transferred to a new tube and centrifuged at 12,000 rpm for 30 min at 4°C to isolate intact crude mitochondria. The cytosolic supernatant was transferred to a new tube and centrifuged two more times to remove any residual mitochondria. Pelleted crude mitochondria were resuspended and washed four times in ice-cold SEM buffer and immediately flash frozen. Mitochondrial pellets were resuspended in 3X packed-cell volume of ice-cold lysis buffer (20mM Tris-HCl pH 7.5, 150mM NaCl, 1mM EDTA, 1% (v/v) NP-40, 2mM sodium orthovanadate, 10mM beta-glycerophosphate, 1X cOmplete protease inhibitor cocktail (Roche)). Cell pellets were homogenized by repeat pipetting, then mixed for 30 minutes at 4°C, followed by centrifugation at 20,000 x g for 15 min.

Western blotting

Samples were subjected to gel-electrophoresis on NuPage 3-8% Tris Acetate (Invitrogen) pre-cast mini-gels with 1X MOPs buffer (Invitrogen) for 1 hour at 150V. Separated proteins were transferred onto a methanol-activated PVDF membrane (Pall or Bio-Rad) for 90 minutes at 100V in 1X transfer buffer (Invitrogen) with 20%(v/v) methanol. Membranes were blocked with either 2% (w/v) skim milk in PBST or ReliaBLOT BLOCK (Bethyl) in TBST for 1 hour at room temperature prior to incubation with primary antibodies at 4°C overnight. For protein signal detection, membranes were incubated with goat anti-mouse HRP-IgG (dilution 1:5000, Santa Cruz), goat anti-rabbit HRP IgG (dilution 1:5000, Santa Cruz), or ReliaBLOT anti-Rabbit HRP (dilution 1:5000, Bethyl) for 1 hour at room temperature followed by three PBST washes before application of either full-strength ECL substrate (GE Healthcare or Bio-Rad) or SuperSignal West Femto substrate (Thermo Scientific). Images were captured using a LAS-3000 imager (FujiFilm) or ChemiDoc™ MP Imager (Bio-Rad). Protein quantitations were performed either using Image Quant 5.1 software or Image Lab 4.1 (Bio-Rad) software.

Mass Spectrometry, MRM and MS/MS analysis:

For each sample, 3mg of total protein was used for immunoprecipitation and preparation for mass-spectrometry. For MS/MS, captured protein samples were separated by size on 3-8% NuPage Tris-Acetate mini-gels in 1X Tris-Acetate running buffer (Invitrogen) by gel-electrophoresis at 150V for 1.5 hours at room temperature. For size determination of endogenous CIC isoforms by MRM, immunoprecipitated proteins were separated by size on 3-8% NuPage Tris-Acetate mini-gels at 150V for 4 hours at 4ºC. Gels were stained with 1X Coomassie blue (20% ethanol, 1.6% phosphoric acid, 8% ammonium sulfate, 0.08% Coomassie Brilliant Blue G-250) overnight at room temperature. After destaining

in 10% v/v methanol at room temperature, protein bands were excised using Hi-Mark pre-stained markers (Invitrogen) as a guide, and digested with trypsin. MRM mass spectrometry was performed as described elsewhere [4]. Peptides used to detect CIC isoforms can be found in Supplementary Figure 2. For identification of CIC interaction candidates by LC-MS/MS the digested protein bands from the gel were analyzed on a 4000 QTrap mass spectrometer (Applied Biosystems/Sciex, Foster City, CA, USA) operating in a data dependent acquisition mode. In this mode of operation each cycle is composed of a full MS scan (400-1600m/z) followed by five MS/MS scans of the most abundant peptides. The acquired MS/MS spectra from each gel lane were concatenated and searched against the Uniprot human database using Mascot (Matrix Science, Boston, MA, USA) search engine. Candidate interacting proteins were those seen in at least two experimental sample replicates and not in the controls and had at least 2 peptides and a Mascot score greater than 50.

Multiple-reaction monitoring (MRM) of metabolites:

Cells were plated in a 10-cm tissue culture plates at a density of $5x10^6$ cells for each stable cell line on the day prior to extraction. Extraction was performed as described in [5] with some modifications. Two hours prior to extraction, culture media was replaced with fresh media. Cells were washed once with PBS, trypsinized and collected in media in 15-mL conical tubes. An aliquot was reserved for cell counts. Cells were centrifuged and the collected cell pellet was immediately resuspended in 3.5 mL of cold 85:15 methanol:water (freshly prepared and stored at -80ºC until use) and immediately frozen at -80ºC for one hour to simultaneously lyse cells and quench metabolism. The cell lysate/methanol mixture was centrifuged at 18,514g for 10 min at 4ºC, and the supernatants were collected and centrifuged to remove cell debris. Supernatants were dried under vacuum using a speedvac and stored at -80C. For protein removal, metabolite pellets were resuspended in ddH2O and filtered through C18 (Empore, 3M, MN, USA) Stagetips [6] that were found to be non-retentive for the polar metabolites examined here. Isolates were collected in standard 96 well plates (Axygen, MA, USA) and dried by vacuum centrifugation. Isolates were suspended in 40 uL ddH2O and 8uL of this material was used for each sample injection. HPLC separations were performed using an Agilent (CA, USA) 1100 HPLC equipped with an autosampler (maintained at 4C). Chromatographic separations described here were performed on commercial C18 columns (Kinetex 2.6um, 15cm x 4.6mm, part# 00f-4462-E0, Phenomenex, Torrance, CA, USA). Separations were conducted with a mobile phase gradient of 100% water to 30% acetonitrile over 5 minutes with a flow rate of 350uL/min. HPLC separated metabolites were interfaced to a 4000QTRAP (ABSciex, Concord, Canada) mass spectrometer with the ion source potential maintained at -3.0 kV. The assays described here were originally optimized by the direct infusion of commercial standards (>95% purity). Under these conditions, optimal QTRAP MRM settings were determined to be:

Q1	Q3	Dwell	Compound	Decluster	Collision Energy	CXP
147.0	103.0	150	Hydroxyglutate	-50	-20	-7
147.0	129.0	150	Hydroxyglutate*	-50	-16	-7
191.0	85.0	200	Citrate/Isocitrate*	-26	-30	-10

191.1	117.0	200	Citrate/Isocitrate	-50	-20	-15
145.0	101.0	200	α-Ketoglutarate*	-50	-18	-4
87.0	59.0	200	Pyruvate*	-36.5	-12.8	-8.9
130.9	69.8	200	Oxaloacetate*	-13.8	-25.14	-10.4
133.3	114.9	200	Malate*	-44.2	-16.22	-8
133.3	71.1	200	Malate	-44.2	-21.9	-10
91.2	59.0	200	Glycerol*	-50.6	-15.13	-5
91.2	88.7	200	Glycerol	-50.6	-9.7	-5
89.1	43.1	150	Lactate*	-46.4	-18	-15

*Metabolite transition used for quantification. Dwell time in milliseconds (msec), Q1/Q3 in mass-to-charge (*m/z*), decluster, collsion energy, CXP (collision exit potential) are in units of relative potential.
Quantification was obtained by determining peak area using integration functions contained within the MultiQuant MRM analysis package (v.1.1 or 2.1, AB Sciex); for more details please see Supplemental Data S2.

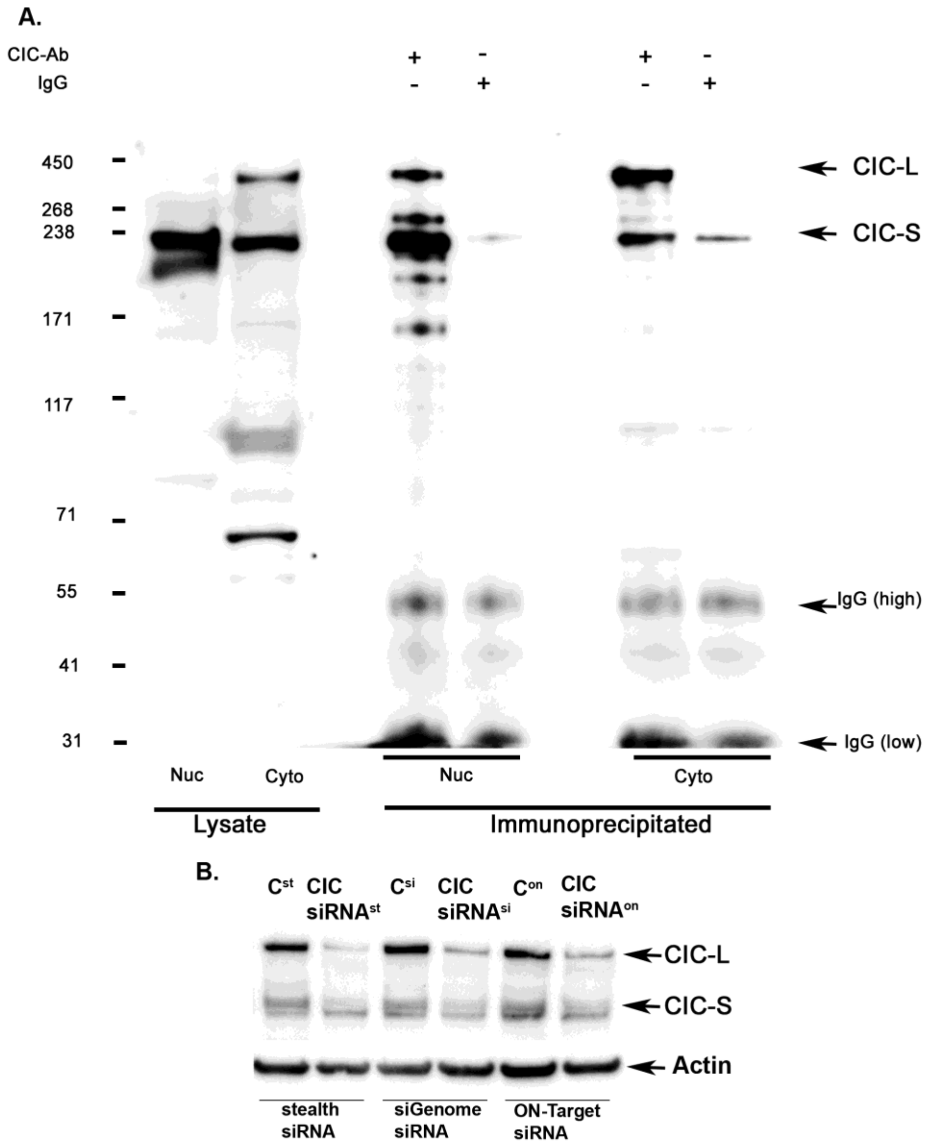

Figure S1: Western blot analysis of CIC isoforms. A. Nuclear and cytoplasmic fractions were isolated from HEK cells, immunoprecipitated with anti-CIC antibody (A301-203A (Bethyl Laboratories) and the protein bands were detected using a different anti-CIC antibody (A301-204A (Bethyl Laboratories) and western blot analysis.. The data shown are representative of at least three independent experiments. **B.** HEK cells were transfected with a stealth siRNA (siRNA1; siRNAst), siGenome (siRNA^{si}) or ON-Targer siRNA (siRNA^{on}) against CIC with the supplied controls. Reductions in CIC-S and CIC-L were detected after 72hrs using western blots. Data are representative of three independent experiments.

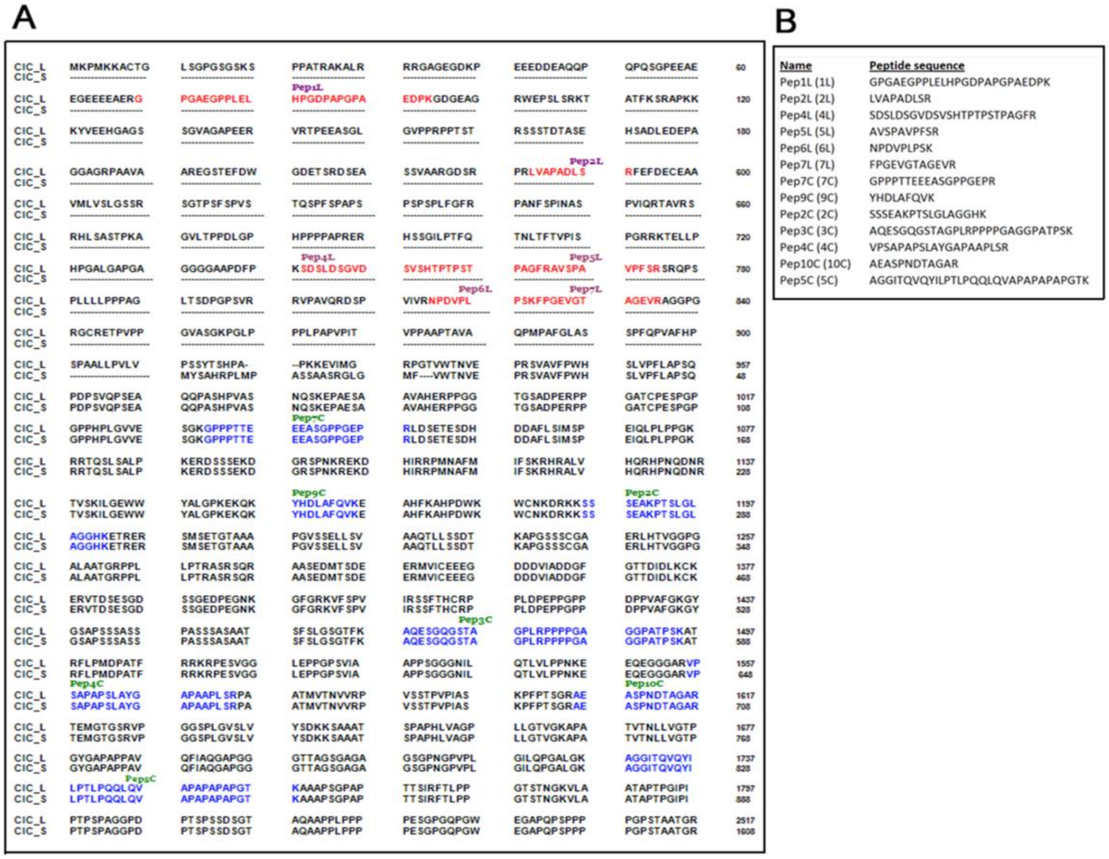

Figure S2: A protein sequence alignment of CIC-L and CIC-S showing peptides designed for MRM. A. A protein sequence alignment of CIC-L and CIC-S. Peptide sequences specific to CIC-L are shown in red and peptide sequences common to both isoforms are shown in blue. Note that only the regions encompassing the peptides are shown; full length CIC-L and CIC-S are not shown. **B.** A table showing sequences of each peptide designed for MRM experiment.

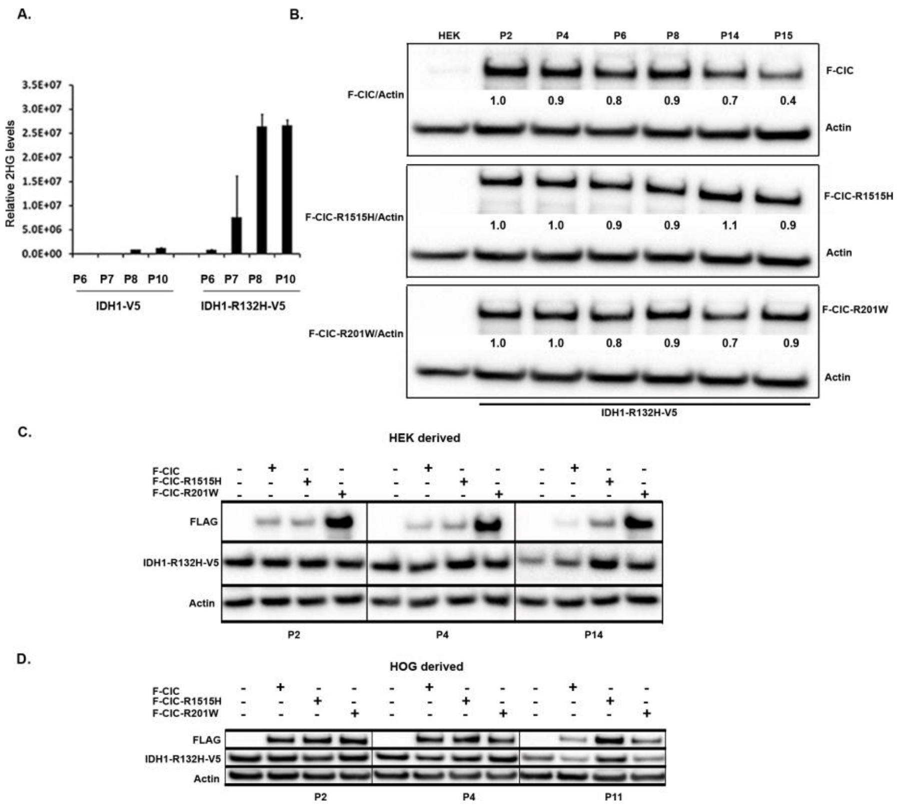

Figure S3: In late passages, 2-HG levels accumulate and mutant CIC proteins are stable. A. Levels of total cellular 2HG from four consecutive passages (P6, P7, P8 and P10) were detected in stably transfected lines expressing IDH1-V5 or IDH1-R132H-V5 using mass spectrometry. 2HG levels continued to increase up to passage 8, after which stabilization of 2HG levels were observed. Shown is the peak area of MRM transitions for 2HG per million cells. Error bars represent the SD from 3 independent MS runs of the same sample. **B.** Stable cell lines co-expressing wild type or mutant IDH1 with wild type or mutant CIC proteins were detected over consecutive passages (P2-P15) using anti-FLAG antibody. Levels of expressed CIC protein relative to Actin (control) was determined (CIC/Actin ratio). Over passages, a gradual decrease in wild type F-CIC was evident (data shown are from cells expressing mutant IDH1-R132H) however, mutant CIC (F-CIC-R1515H (or –R201W) proteins were stable. C. Samples from early (eg. P2, P4) and late passages (eg. P14) from HEK stable lines (see B) expressing mutant IDH1 were run side by side and CIC (FLAG) or IDH1 (V5) proteins were detected. While levels of F-CIC and F-CIC-R1515H were comparable after selection (P2), F-CIC-R201W expression was higher. IDH1-R132H levels were comparable in early passages however, in late passages, the highest level of IDH1-R132H was observed in cells also expressing F-CIC-R1515H. D. Samples from early (eg. P2, P4) and late passages (eg. P11) from HOG stable lines expressing mutant IDH1 were run side by side and CIC (FLAG) or IDH1 (V5) proteins were detected. In HOG-derived stable lines, levels of F-CIC, F-CIC-R1515H and F-CIC-R201W were comparable in early passages immediately after selection (P2, P4), but F-CIC-R1515H expression was the highest in late passages (P11). IDH1-R132H levels were comparable in early passages but,in late passages, the highest level of IDH1-R132H was observed in cells also expressing F-CIC-R1515H.

References:

1. Fujiki Y, Hubbard AL, Fowler S, Lazarow PB. Isolation of intracellular membranes by means of sodium carbonate treatment: application to endoplasmic reticulum. J. Cell Biol. 1982; 93(1):97–102.

2. Dissanayake K, Toth R, Blakey J, Olsson O, Campbell DG, Prescott AR, MacKintosh C. ERK/p90(RSK)/14-3-3 signalling has an impact on expression of PEA3 Ets transcription factors via the transcriptional repressor capicúa. Biochem. J. 2011; 433(3):515–525.

3. Lam YC, Bowman AB, Jafar-Nejad P, Lim J, Richman R, Fryer JD, Hyun ED, Duvick LA, Orr HT, Botas J, Zoghbi HY. ATAXIN-1 interacts with the repressor Capicua in its native complex to cause SCA1 neuropathology. Cell 2006; 127(7):1335–1347.

4. Yip S, Butterfield YS, Morozova O, Chittaranjan S, Blough MD, An J, Birol I, Chesnelong C, Chiu R, Chuah E, Corbett R, Docking R, Firme M, Hirst M, Jackman S, Karsan A et al. Concurrent CIC mutations, IDH mutations, and 1p/19q loss distinguish oligodendrogliomas from other cancers. J. Pathol. 2012; 226(1):7–16.

5. Yuan M, Breitkopf SB, Yang X, Asara JM. A positive/negative ion-switching, targeted mass spectrometry-based metabolomics platform for bodily fluids, cells, and fresh and fixed tissue. Nat. Protoc. 2012; 7(5):872–881.

6. Rappsilber J, Ishihama Y, Mann M. Stop and go extraction tips for matrix-assisted laser desorption/ionization, nanoelectrospray, and LC/MS sample pretreatment in proteomics. Anal. Chem. 2003; 75(3):663–670.
